# Dexamethasone as a Modulator of Renin–Angiotensin System Receptor Expression in Prostate and Ovarian Cancer Cells Under Standard and Low-Serum Conditions

**DOI:** 10.3390/cancers18121998

**Published:** 2026-06-19

**Authors:** Weronika Broszkiewicz, Natasza Wiertek-Płoszaj, Katarzyna Gajewska, Anna Wosiak, Kamila Domińska

**Affiliations:** Department of Comparative Endocrinology, Medical University of Lodz, Zeligowskiego 7/9, 90-752 Lodz, Poland; weronika.broszkiewicz@umed.lodz.pl (W.B.); natasza.wiertek-ploszaj@umed.lodz.pl (N.W.-P.);

**Keywords:** dexamethasone, glucocorticoid receptor, ovarian cancer, prostate cancer, serum deprivation, *AGTR1*, *AGTR2*, *MAS1*, *LNPEP*, RAS

## Abstract

**Background/Objectives**: Glucocorticoids, including dexamethasone (DEX), are known to demonstrate anti-inflammatory activity, suppress steroidogenesis, and mitigate the adverse effects of chemotherapy. They are therefore widely employed for managing solid malignancies. Emerging evidence indicates that DEX modulates both systemic and local renin–angiotensin system (RAS) activity, including genomic signaling via the glucocorticoid receptor (GR). **Methods**: DEX-dependent transcriptional responses for the angiotensin receptor genes (*AGTR1*, *AGTR2*, *MAS1*, and *LNPEP*) were evaluated in ovarian (SKOV3, KURAMOCHI) and prostate (DU-145, PC3) cancer cell lines. The cells were cultured under different serum conditions to determine the influence of nutrient availability on tumor progression. **Results**: DEX demonstrated distinct mechanisms of action between the ovarian and prostate cancer models. It was found to promote cancer cell survival through tissue-specific modulation of metabolic activity, clonogenic capacity, cell cycle distribution, and apoptotic responses. These effects were accompanied by condition-dependent alterations in angiotensin receptor gene expression. Hence, DEX may mediate the remodeling of local RAS signaling, which may be significant in overall survival and disease-free survival. The findings also indicate a previously-unreported *NR3C1–LNPEP* correlation, which was consistently observed across in vitro systems and patient datasets, in both ovarian- and prostate-derived cancer models. **Conclusions**: DEX appears to exert context-dependent regulation of RAS-associated gene networks in ovarian and prostate cancer, suggesting a role in tumor adaptive responses and potentially in therapeutic contexts.

## Simple Summary

In this study, we evaluated the effect of dexamethasone on the expression of local renin–angiotensin system receptors in prostate and ovarian cancer cells cultured under standard conditions or with limited serum availability. Long-term exposure to dexamethasone induced adaptive changes suggesting enhanced cancer cell survival under stress conditions. These changes included alterations in cell-cycle progression, metabolic reprogramming, and reduced apoptosis, depending on the experimental model and serum availability. We propose that such mechanisms may support cancer cell adaptation and survival under stress conditions and potentially influence treatment response. The results further identify a consistent correlation between *NR3C1* and *LNPEP* expression across ovarian and prostate cancer models as well as patient datasets, suggesting potential crosstalk between glucocorticoid receptor signaling and the renin–angiotensin system.

## 1. Introduction

The effects of glucocorticoids (GCs) on the renin–angiotensin system (RAS) have been known since the late 1970s [1], with its underlying systemic and tissue-level mechanisms becoming elucidated over time. The majority of research into interactions between GC and RAS concern the regulation of fluid and electrolyte balance (e.g., hypokalemia, hypernatremia) [2] and cardiovascular function (e.g., hypertension, left ventricular hypertrophy, heart failure) [3,4]. However, the interaction has also been found to play a role in bone metabolism (e.g., osteoporosis, osteonecrosis) [5], renal function (e.g., reduced glomerular filtration, renal fibrosis) [6], and pulmonary health (e.g., susceptibility to SARS-CoV-2 infection, course of COVID-19) [7]. One highly potent synthetic GC, dexamethasone (DEX), has been shown to modulate RAS activity at multiple levels. It has been found to influence the expression of genes encoding key components of the RAS, including angiotensin receptors and enzymes involved in angiotensin synthesis and metabolism [6,8]. In addition, DEX may affect RAS activity through several physiological pathways, including hemodynamic regulation [8], ion transport and local hormonal signaling [6]. Given the widespread and often prolonged use of DEX across medical fields, it is essential to understand its effects on the RAS and to identify any associated adverse outcomes of treatment.

In addition to their systemic effects, GC–RAS interactions may also play a role in cancer biology. While GCs, such as DEX and hydrocortisone, possess anti-inflammatory activity and are able to suppress steroid hormone synthesis, they have also been found to mitigate the adverse effects of chemotherapy, including nausea, vomiting, edema, and hypersensitivity reactions [9,10]. As such, they are also routinely employed as adjunctive agents in the management of solid tumors, including prostate and ovarian cancer. However, mounting evidence suggests that GC may increase the risk of relapse by supporting the persistence of minimal residual disease [11,12].

The solid tumor microenvironment is often subject to nutrient deprivation, primarily resulting from limited and/or competing blood supply [13]. In ovarian cancer, glucocorticoid administration has been associated with upregulation of anti-apoptotic genes SGK1 and MKP1/DUSP1 in tumor tissue [14]. Dexamethasone acts as a cytostatic agent in prostate cancer cells, inhibiting cell proliferation and enhancing the G0/G1 cell-cycle arrest induced by serum deprivation. However, it also suppresses apoptosis and promotes metabolic adaptation and cell survival under stress conditions [15].

The RAS has emerged as an important regulator of tumor development and progression. The classical ACE/Ang II/AT1 axis is generally associated with pro-tumorigenic effects, including enhanced proliferation, angiogenesis and cell migration, whereas the counter-regulatory ACE2/Ang-(1–7)/Mas/AT2 axis limits tumor progression by controlling anti-proliferative and pro-apoptotic signaling [16].

The present study examines whether DEX influences prostate and ovarian cancer cells through the modulation of the local RAS. Currently, the effects of DEX on the expression of local RAS receptors in these cancer types remain unstudied. To complement the experimental findings, the study also presents a bioinformatic analysis of glucocorticoid receptor and renin–angiotensin system-related gene expression, including co-expression analyses and comparisons with clinical outcome data.

## 2. Materials and Methods

### 2.1. Cell Culture and Reagents

Two ovarian cancer cell lines were acquired for the study. The first was the widely used SKOV3 cell line (American Type Culture Collection, Manassas, VA, USA). According to recent molecular analyses, this cell line exhibits features consistent with clear cell ovarian carcinoma (OCCC) rather than high-grade serous ovarian carcinoma (HGSOC) [17]. The second was the less widely known KURAMOCHI cell line, obtained from the JCRB Cell Bank (Japanese Collection of Research Bioresources Cell Bank, Osaka, Japan), which is considered a model of HGSOC and reflects key molecular characteristics of this subtype. These two were accompanied by two human prostate cancer cell lines: the DU-145 line, derived from a central nervous system metastasis (American Type Culture Collection; Manassas, VA, USA), and the PC3 line, originating from a bone metastasis (European Collection of Authenticated Cell Cultures; Porton Down, Salisbury, UK). The prostate cancer cell lines were selected as androgen-independent models, which are known to rely on alternative signaling pathways, including GR-mediated signaling.

All cell lines grow adherently, exhibit epithelial morphology, and express glucocorticoid receptor. However, DU-145 cells exhibit higher GR expression than PC3 cells at the mRNA and protein levels. Similarly, in the ovarian cancer cell lines, SKOV3 cells exhibit higher glucocorticoid receptor gene (*NR3C1*) expression than KURAMOCHI cells [18,19]. 

The cell lines were authenticated by short tandem repeat (STR) profiling (LGC Standards Cell Line Authentication Service, Germany; 2019/2021). Passages that were directly frozen after authentication were used for experiments. The human ovarian and prostate cancer cell lines were cultured in McCoy’s 5A medium (SKOV3) or RPMI-1640 medium (KURAMOCHI, DU145 and PC3) supplemented with the following: 10% heat-inactivated fetal bovine serum (FBS) and standard additives, including L-glutamine, HEPES, sodium pyruvate and penicillin-streptomycin. Incubation was performed at 37 °C with 5% CO_2_ and 95% humidity. Cell culture medium and supplements were purchased from Gibco (Thermo Fisher Scientific, Inc., Waltham, MA, USA).

Dexamethasone was purchased from Merck (Sigma-Aldrich, Saint Louis, MO, USA). The stock solution (20 µg/mL) was prepared according to the instructions by adding 1 mL of absolute ethanol and 49 mL of sterile medium. Experimental DEX dilutions (100 nM or 10 nM) were prepared in medium containing either the full serum concentration (10% FBS) or a reduced serum concentration (5% FBS, mild serum starvation conditions). The incubation period included two time points: 72 h (three days) and 192 h (nine days). The experimental procedures were adapted from previous work in prostate cancer cells [15]. The final dexamethasone concentrations were selected based on preliminary optimization experiments, reproducibility of biological responses, and previously published studies investigating glucocorticoid signaling in cancer models [19,20,21]. The selected concentrations also approximate clinically relevant plasma dexamethasone levels reported in pharmacokinetic studies, ranging from standard therapeutic to higher pharmacological exposure conditions [22,23].

### 2.2. Metabolic Activity-Based Assays (MTT and Alamar Blue Assay)

Ovarian cancer cells were seeded in 12-well plates at densities adjusted according to the serum concentration and the incubation period. Twenty-four hours after seeding, the culture medium was replaced with an experimental medium (10% or 5% FBS) treated with 100 nM DEX. The experimental medium was changed every 24 h. Two hours before the end of the incubation, either MTT (0.5 mg/mL) or Alamar Blue (10% *v*/*v*) reagent was added. Changes in cellular metabolic activity were assessed by measuring absorbance at 570 nm and 600 nm using a BioTek microplate reader (Winooski, VT, USA). Results are presented as percentage of untreated cells.

### 2.3. Colony Formation Assay

The ovarian cancer cells were seeded in 12- or 6-well plates. The seeding density was adjusted to the vessel type, serum concentration, and incubation time, to obtain distinct colonies derived from single-cell proliferation. Twenty-four hours after seeding, the medium was replaced with an experimental medium (5% or 10% FBS) containing 100 nM DEX, which was subsequently replaced every 24 h. After the three- or nine-day incubation period, the wells were gently washed with PBS, and the cell colonies were stained with a crystal violet (0.1% in 25% ethanol). The wells were rinsed (3× with water) to remove excess crystal violet and left to dry. The stained cells were photographed and analyzed for colony number and size using ImageJ software v. 1.52a (Wayne Rasband, NIH, Bethesda, MD, USA; http://imagej.nih.gov/ij accessed on 18 May 2026). The crystal violet was then solubilized in 10% acetic acid, and absorbance was measured at 550 nm using a BioTek microplate reader (Winooski, VT, USA). Results are presented as fold change in untreated cells.

### 2.4. Muse Cell Cycle Assay

After incubation with 100 nM DEX, the ovarian cancer cells were trypsinized and processed according to the manufacturer’s protocol. Briefly, the cell pellet was washed with PBS, fixed in cold 70% ethanol, and stored at −20 °C for at least 24 h. For analysis, cells were stained with propidium iodide, which binds to DNA, and treated with RNase to remove RNA. The samples were incubated in the dark at room temperature for 30 min. Cell cycle distribution was then analyzed using the Muse™ Cell Analyzer microcapillary flow cytometer (Millipore, Burlington, MA, USA), with results expressed as the percentage of cells in each phase relative to controls.

### 2.5. Muse Annexin V & Dead Cell Assay

Following treatment with 100 nM DEX, ovarian cancer cells were trypsinized and processed according to the manufacturer’s protocol. The resulting cell pellet, resuspended in FBS-containing medium, was mixed with an equal volume of staining solution containing fluorescently labeled Annexin V and 7-AAD. Annexin V binds to phosphatidylserine exposed on the outer leaflet of the plasma membrane in apoptotic cells, whereas 7-AAD stains cells with compromised membrane integrity. The samples were incubated for 20 min at room temperature in the dark and then analyzed using a Muse™ Cell Analyzer microcapillary flow cytometer (Millipore, Burlington, MA, USA). Results were expressed as the percentage of apoptotic cells relative to untreated controls.

### 2.6. Reverse Transcription Quantitative Real-Time PCR (RT-qPCR)

After incubation, the ovarian cancer cells (100 nM DEX, 3 or 9 days) and prostate cancer cells (10 or 100 nM DEX, 9 days) were harvested for total RNA extraction using TRIzol reagent. The RNA isolation, quality assessment, and reverse transcription procedures are described in detail elsewhere [15]. Quantitative PCR (qPCR) was performed using EvaGreen dye (Biotium, Fremont, CA, USA) on a LightCycler 96 system (Roche, Basel, Switzerland). Primers were designed and validated using NCBI Primer-BLAST (National Institutes of Health (NIH), Bethesda, MD, USA) to ensure specificity, minimize off-target amplification, and optimize amplification efficiency. Detailed information on primer sequences, amplicon sizes, and PCR conditions is provided in the Supplementary Table S1.

The specificity of the qRT-PCR products was confirmed by melting curve analysis. Most reactions showed a single melting peak, indicating specific amplification. Representative melting curves are provided in Supplementary Figure S1. Reactions showing failed amplification, absence of signal, or abnormal melting curves were excluded from further analysis. *RPLP0* and *H3F3A* were used as reference genes due to their stable expression (SD ≤ 0.5; ΔCt ≤ 1.5, Supplementary Table S2). Universal human RNA (Agilent, Santa Clara, CA, USA) served as the calibrator, and a master mix with nuclease-free water was used as the negative control. Analyses were performed using three independent biological replicates, each analyzed in technical duplicate. To verify the applicability of the ΔΔCt (Livak) method, amplification efficiency was assessed for each target and housekeeping gene using standard curves generated from serial dilutions of pooled cDNA representing all cell lines and experimental variants included in the study. A serial dilution of the cDNA samples was prepared using four dilution points (1×, 5×, 25×, 125×). Standard curves were generated by plotting Ct values against the log_10_-transformed template input. Efficiency was determined from the slope of the linear regression within the linear range of the dilution series according to the equation: E = 10^−1/slope^ − 1 [24]. Ct range, standard curve slope, R^2^, and qualitative assay performance assessment for all genes are summarized in Supplementary Table S3. At 100% amplification efficiency, E = 1, corresponding to a two-fold amplification per cycle. Since the amplification efficiencies of majority assays ranged from 98% to 111%, and all showed standard curve linearity of R^2^ > 0.99, they were considered appropriate for relative gene expression analysis using the ΔΔCt/Livak method [24,25].

### 2.7. Bioinformatic Analysis of Gene Expression, Correlation and Clinical Relevance

Transcriptomic associations and potential functional relationships involving selected receptor genes (*AGTR1*, *AGTR2*, *LNPEP*, *MAS1*, and *NR3C1*) were explored using publicly available bioinformatic platforms. Gene expression across cancer cell lines was assessed using the DepMap Portal (Broad Institute, Data Explorer 2.0, 26Q1). The expression of *NR3C1* was compared between cancer cell lines: gene–gene relationships between prostate (DU145, PC3) and ovarian (SKOV3, KURAMOCHI) lines were determined based on pairwise correlations between selected receptor genes. Additionally, the impact of gene knockdown on cell viability was analyzed using DepMap functional dependency datasets [26]. 

Correlation analysis in patient-derived samples was performed using the Gene Expression Profiling Interactive Analysis (GEPIA) web server, based on data from The Cancer Genome Atlas (TCGA). Pearson correlation coefficients were calculated for TCGA-OV (ovarian cancer) and TCGA-PRAD (prostate adenocarcinoma) cohorts. Non-log-transformed expression values were used for statistical calculations, while log-scale transformation was applied for data visualization [27].

Exploratory survival analyses were conducted using the cBioPortal platform (v 7.0.1) [28,29,30]. Kaplan–Meier curves were generated for overall survival (OS), disease-free survival (DFS), and progression-free survival (PFS). Patients were then stratified into groups based on the lowest and highest quartiles of gene expression. Gene expression was defined using mRNA expression z-scores relative to all samples (log RNA-seq V2 RSEM or log microarray data, depending on dataset availability). For prostate and ovarian cancer analyses, all publicly available studies in cBioPortal at the time of access (March 2026) were screened and filtered according to tumor type. The prostate cancer analysis was restricted to cases classified as prostate adenocarcinoma (*n* = 13,296), whereas the ovarian cancer analysis comprised serous ovarian cancer, high-grade serous ovarian cancer, and ovarian cancer cases (*n* = 2562). Unless otherwise stated, all analyses were performed using default parameters.

### 2.8. Statistical Analysis

Statistical analyses were conducted using GraphPad Prism 9 (GraphPad Software, La Jolla, CA, USA). The normality of distribution was confirmed using the Shapiro–Wilk test. Normally distributed data were evaluated using one-way ANOVA followed by Tukey’s post hoc test, whereas non-normally distributed data were analyzed using the Kruskal–Wallis test with Dunn’s multiple comparisons. Results are expressed as mean ± standard deviation (SD) or median with interquartile range (IQR) accordingly. A *p*-value below 0.05 (*p* < 0.05) was considered statistically significant. All experiments were performed with at least three independent replicates (*n* ≥ 3). These conventions are applied throughout the study, unless otherwise specified.

## 3. Results

### 3.1. Effect of Dexamethasone on the Metabolic Activity of Human Ovarian Cancer Cells

**Figure 1:**
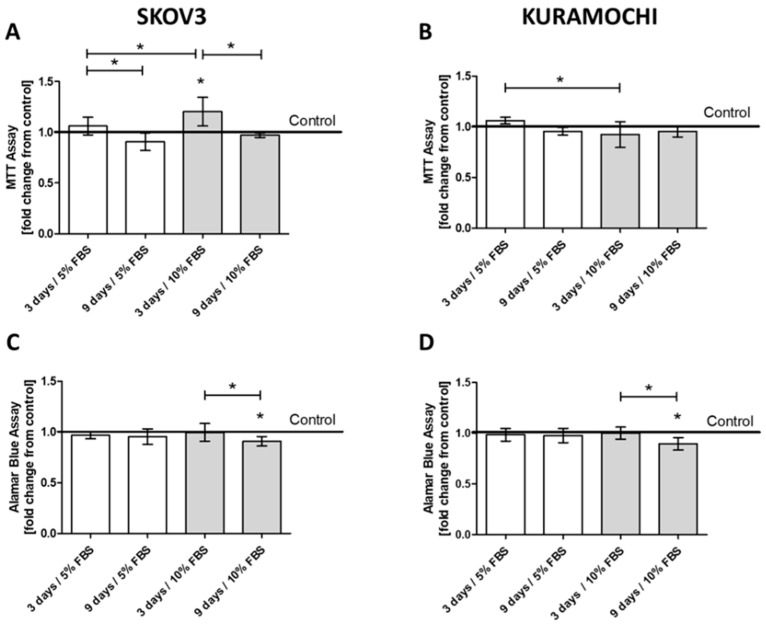
Changes in metabolic activity of SKOV3 and KURAMOCHI ovarian cancer cells following 3- and 9-day DEX treatment (100 nM), expressed as fold change relative to the control and assessed by MTT (**A**,**B**) and Alamar Blue (**C**,**D**) assays. Data are presented as mean ± SD (*n* ≥ 3). One-way ANOVA with Tukey’s post hoc test was used for statistical analysis (* *p* < 0.05).

In SKOV3 cells, a decrease in Alamar Blue reduction was observed after nine days of DEX treatment, although significant changes were only achieved in medium containing 10% serum (Figure 1C). Metabolic activity (MTT assay) decreased following prolonged DEX exposure, whereas a transient increase was observed after short-term treatment. However, statistical significance was reached only for the 3-day treatment in medium supplemented with 10% FBS (Figure 1A). Similarly, in the KURAMOCHI cells, a decrease in Alamar Blue reduction was observed only after nine days of treatment in medium containing 10% FBS (Figure 1D). In the three-day treatment variant, higher metabolic activity (MTT score) was associated with mild serum starvation, and lower activity with higher serum content. However, none of these changes were significantly different to controls (Figure 1B).

### 3.2. Effect of Dexamethasone on the Colony Formation of Human Ovarian Cancer Cells

**Figure 2:**
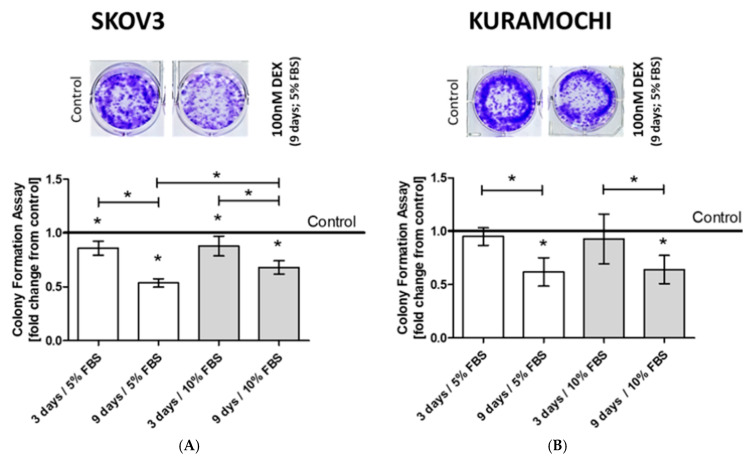
Fold change in colony formation relative to the control in ovarian cancer cells following 3- and 9-day treatment with DEX (100 nM) ((**A**), SKOV3; (**B**), KURAMOCHI). Data are presented as mean ± SD (*n* ≥ 3). Statistical analysis was performed using one-way ANOVA followed by Tukey’s post hoc test (* *p* < 0.05).

In SKOV3 cells, DEX treatment triggered a marked reduction in cell number after just three days, with the effect increasing with longer exposure (Figure 2). The inhibitory effect was dependent on the serum concentration in the experimental medium, being less pronounced in 10% FBS than in 5% FBS. In KURAMOCHI cells, a measurable decrease in clonogenic capacity was observed only after prolonged DEX exposure, irrespective of serum concentration (Figure 2).

### 3.3. Effect of Dexamethasone on Cell Cycle Distribution in Human Ovarian Cancer Cells

**Figure 3:**
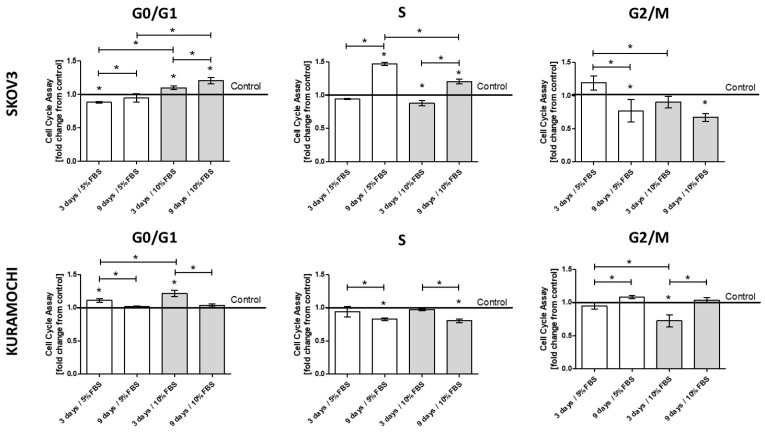
Cell cycle distribution (G0/G1, S, and G2/M phases) of SKOV3 and KURAMOCHI ovarian cancer cells following 3- and 9-day treatment with DEX (100 nM). Data are expressed as fold change relative to the control and presented as mean ± SD (*n* ≥ 3). One-way ANOVA followed by Tukey’s post hoc test was used for statistical analysis (* *p* < 0.05).

In SKOV3 cells cultured in 10% FBS, DEX treatment increased the proportion of cells in the G0/G1 phase regardless of treatment duration. In contrast, under 5% FBS conditions, the G0/G1 fraction decreased, reaching statistical significance only after 3 days of treatment (Figure 3). The S-phase population was strongly dependent on exposure time: the number of cells fell after three days (significant only in 10% FBS) but increased after nine days. The G2/M population fell after nine days, regardless of serum concentration, while it increased after three days under mild serum starvation.

In KURAMOCHI cells, short-term DEX exposure increased the G0/G1 population, with a stronger effect noted in 10% FBS. A significant decrease in S-phase cells was observed after nine days of treatment, irrespective of FBS concentration. In the G2/M phase, differences were noted with controls only after three days in 10% FBS; however, a marked increase was observed after extending DEX exposure from three to nine days.

### 3.4. Effect of Dexamethasone on Apoptotic Regulation in Human Ovarian Cancer Cells

**Figure 4:**
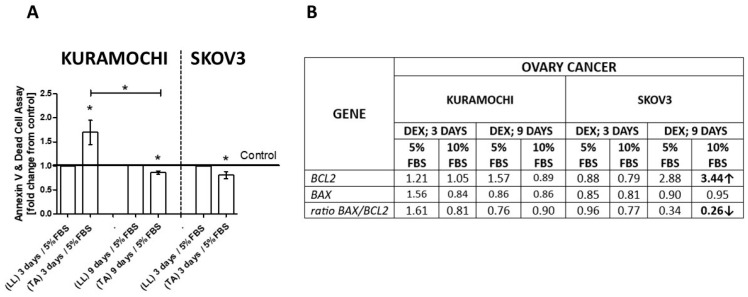
Analysis of apoptosis (**A**) and *BCL2*/*BAX* expression (**B**) in DEX-treated human ovarian cancer cells (100 nM). Apoptosis data are presented as mean ± SEM (*n* ≥ 3), while gene expression is shown as median fold change relative to control. Arrows indicate statistically significant changes: ↓—decrease; ↑—increase (one-way ANOVA with Tukey’s test or Kruskal–Wallis test with Dunn’s multiple comparisons: * *p*  <  0.05) (LL, live cells; TA, total apoptotic cells).

In KURAMOCHI cells, short-term DEX treatment (three days) under serum starvation (5% FBS) increased apoptosis, whereas prolonged exposure (nine days) suppressed it. In addition, while no significant change in apoptotic gene expression (*BCL2* and *BAX*) was noted, the *BAX*/*BCL2* ratio was found to increase after three days of DEX treatment and decrease after nine days. These findings are consistent with those of the Muse Annexin V & Dead Cell Assay (Figure 4B).

In SKOV3 cells, apoptosis was significantly reduced after just three days of DEX treatment (Figure 4A). Under 10% FBS, nine-day exposure led to a significant increase in *BCL2* expression and a decrease in the *BAX*/*BCL2* ratio; a similar trend was observed for the 5% FBS variant, although these changes were not significant.

### 3.5. Expression of Angiotensin and Glucocorticoid Receptors in Prostate and Ovarian Cancer Cells and Their Modulation by Dexamethasone

**Figure 5:**
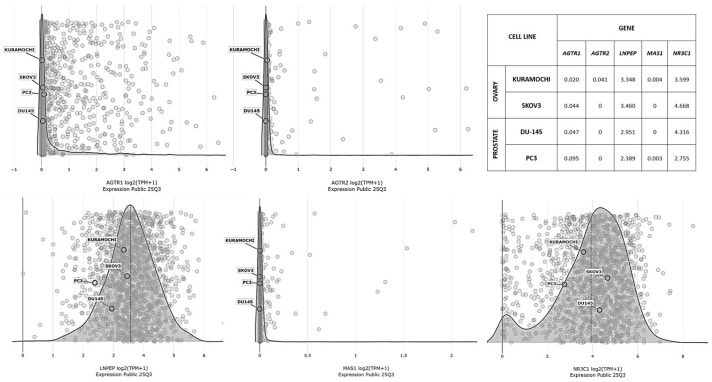
Expression levels of selected genes (*AGTR1*, *AGTR2*, *LNPEP*, *MAS1* and *NR3C1*) in KURAMOCHI, SKOV3, DU145, and PC3 cell lines based on DepMap data. Expression values are shown as log2(TPM + 1). TPM (Transcripts Per Million) represents the normalized abundance of RNA transcripts. The dashed vertical line indicates the median gene expression level within each cell line group.

The analysis of the DepMap data indicated that the expression of the *AGTR2* and *MAS1* was generally below the detection threshold across the analyzed cell lines. All cell lines exhibited *AGTR1* expression, although at low levels. Among the angiotensin receptors, the highest expression was observed for *LNPEP*, with higher levels in ovarian cancer compared with prostate cancer cells. The *NR3C1* was expressed in all studied cell lines; in the ovarian cancer lines, higher expression was observed in SKOV3 than in KURAMOCHI, while in the prostate cancer lines, higher expression was noted in DU-145 than in PC3 (Figure 5).

Dexamethasone treatment modulated expression of angiotensin-related genes in a cell line– and condition-dependent manner. In KURAMOCHI cells, AGTR1, MAS1 and LNPEP showed increased mRNA levels after prolonged incubation (9 days) under low-serum conditions (5% FBS). In SKOV3 cells, exposure to DEX in complete growth medium (10% FBS) significantly upregulated AGTR1 and LNPEP, while, AGTR2 and MAS showed only non-significant trends toward increased expression after prolonged exposure, particularly under 10% FBS conditions. In the AGTR2 and MAS1 assays, compression of the standard curves was observed at mid-to-high Ct values, indicating reduced linearity and suggesting that quantification in this range should be interpreted with caution. Overall, in SKOV3 cells, short-term DEX exposure tended to reduce angiotensin receptor expression, whereas prolonged treatment showed a trend toward increased expression, although these changes were not statistically significant. In androgen-independent prostate cancer cells, dexamethasone decreased LNPEP expression, with statistically significant changes observed only in DU-145 cells (Table 1).

In KURAMOCHI cells, GR mRNA levels significantly increased after nine days of DEX exposure under 5% FBS but decreased under 10% FBS. No differences in *NR3C1* expression were observed in SKOV3 cells. In all prostate cancer cells, DEX exposure was associated with a downward trend in GR gene expression; however, these changes were not statistically significant (Table 1).

### 3.6. Biological Roles and Interrelationships of Angiotensin and Glucocorticoid Receptors in Prostate and Ovarian Cancer Cells

Data from prostate and ovarian cancer patients available in cBioPortal were analyzed to evaluate overall survival (OS), disease-free survival (DFS), and progression-free survival (PFS) according to the expression levels of glucocorticoid receptor and renin–angiotensin system-related genes. (Table 2). OS indicates survival accounting for all causes of death, DFS measures the time free of disease recurrence after treatment, allowing the effects of therapy to be distinguished from the natural course of the disease, and PFS focuses on the time to disease progression, regardless of recurrence [31].

In ovarian cancer, high *AGTR1* expression was associated with poorer OS and DFS, while *AGTR2* expression was associated with improved DFS. Neither relationship was observed in prostate cancer. *LNPEP* and *MAS1* expression were inversely associated with OS in ovarian cancer. In prostate cancer, low *LNPEP* expression was associated with poorer DFS, whereas *MAS1* expression was not associated with any survival endpoint after correction for multiple testing. *NR3C1* expression showed positive associations with OS, DFS, and PFS in prostate cancer, with higher expression corresponding to improved patient outcomes. In ovarian cancer, only a weak, non-significant inverse trend was observed (Table 2, Supplementary Figures S2 and S3).

The impact of gene knockouts on the viability of the studied cells was assessed using DepMap data. None of the analyzed genes were essential for the survival of the studied cells. Weak effects were noted for *AGTR1* in DU-145 and *MAS1* in PC3, with scores slightly above −0.25 (dependency scores) (Supplementary Figure S4).

**Figure 6:**
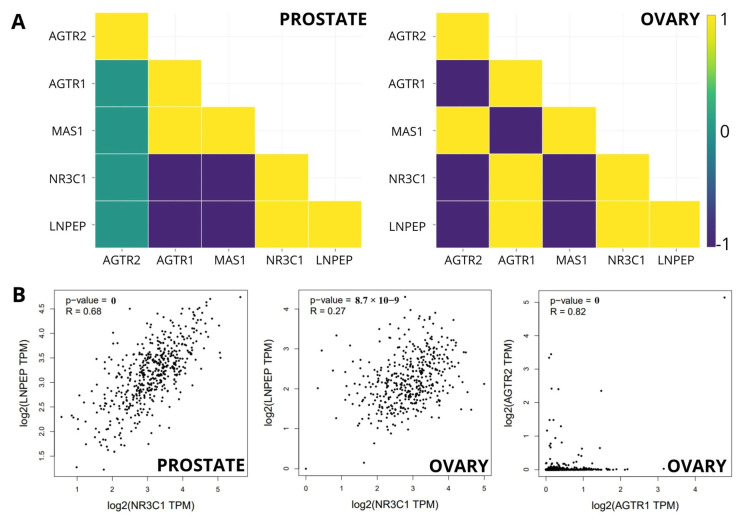
The expression of RAS components genes (*AGTR2*, *AGTR1*, *MAS1*, *LNPEP*) and the glucocorticoid receptor (*NR3C1*) were analyzed in prostate and ovarian cancer models. Correlation heatmaps (**A**) were generated using DepMap data to visualize gene expression patterns in cancer cell lines (SKOV3, KURAMOCHI, DU-145, and PC3), whereas scatter plots of significant relationships (**B**) were based on patient-derived data obtained from GEPIA.

To identify potential links between the RAS and GC signaling in cancer, the expression of the *AGTR1*, *AGTR2*, *MAS1* and *LNPEP* genes were compared with that of the glucocorticoid receptor *NR3C1*. To identify consistent patterns of co-regulation between cell lines, analyses were performed in both the prostate (DU-145, PC3) and ovarian cancer (SKOV3, KURAMOCHI) cell lines, as well as in patient-derived datasets. In the prostate and ovarian cancer cell lines, *LNPEP* expression was positively correlated with *NR3C1* (Figure 6A). Further analysis revealed that the relationship between *MAS1* and *AGTR1* was positive in prostate cancer lines but negative in ovarian cancer lines. In the same dataset, *AGTR2* showed no significant correlations in prostate cancer, whereas in ovarian cancer it was positively correlated with *MAS1* and negatively correlated with *AGTR1*, *LNPEP*, and *NR3C1*.

The patient-derived data revealed significant associations between *NR3C1* and *LNPEP* in both ovarian and prostate samples, as well as between *AGTR1* and *AGTR2* in ovarian samples. The correlation between *NR3C1* and *LNPEP* mirrored the patterns observed in cell lines; in contrast, a strong positive correlation was observed between *AGTR1* and *AGTR2* (R = 0.82), although most ovarian samples exhibited low expression of both genes (Figure 6B, Supplementary Figures S5 and S6).

## 4. Discussion

Studies on prostate cancer cells under mild serum deprivation found prolonged exposure to DEX (10–100 nM) to reduce metabolic activity, as evidenced by decreased MTT and resazurin reduction [15]. In contrast, in the present study, the ovarian cancer cells exhibited a distinct, assay-dependent response profile: after prolonged exposure (nine days) under full-serum conditions, the SKOV3 and KURAMOCHI cells demonstrated more apparent changes in metabolic activity in the Alamar Blue assay than the MTT assay (Figure 1). These differences likely reflect the distinct biochemical principles underlying the applied assays, as MTT predominantly measures mitochondrial dehydrogenase activity, whereas Alamar Blue reflects broader cellular redox activity [32]. They also derive from the distinct metabolic properties of the assays and the glycolytic phenotype of ovarian cancer cells [33]; as ovarian cancer cells tend to demonstrate relatively low mitochondrial activity, DEX-induced changes are less apparent in MTT assays compared to Alamar Blue.

Despite these differences in the observed metabolic profile, both ovarian and prostate cancer [15] cells showed a reduction in clonogenicity following DEX exposure, suggesting a cytostatic effect (Figure 2). In the SKOV3 cells, this effect was observed as early as three days and was more pronounced than in the KURAMOCHI cells, which required prolonged exposure. The magnitude of this response may be related to differences in glucocorticoid receptor (*NR3C1*) expression between cell lines (Figure 5). In ovarian cancer cells, crystal violet staining revealed a reduction in total cell number, whereas metabolic activity assays did not show a corresponding decrease. This effect was not observed in previous studies on prostate cancer cells, potentially reflecting differences in cellular metabolic programs across cancer types [34]. Glucocorticoids have been reported to influence mitochondrial function, affecting both mitochondrial respiration and oxidative and reduction enzyme activity via nuclear and mitochondrial GRs; however, it should be noted that the direction and magnitude of these effects largely depend on cell type, dose, and duration of exposure [35].

The cytostatic effects observed following DEX exposure were accompanied by alterations in cell cycle distribution. In prostate cancer cells, prolonged exposure resulted in G0/G1 arrest, as noted previously [36]. In ovarian cancer cells, the response depended on both cell line and exposure time. Short-term treatment was generally associated with an increase in the G0/G1 population, whereas prolonged exposure led to distinct changes in the S phase (Figure 3).

In the ovarian cell lines, after nine days, the S-phase cell population increased in SKOV3 cells but decreased in KURAMOCHI cells; this change was accompanied by a decrease in G2/M in SKOV3. Hence, DEX appears to modulate cell cycle progression in a context-dependent manner rather than inducing uniform arrest across models. In SKOV3 cells, progression through the S phase appears to be slowed and entry into G2/M appears to be delayed. This may allow the cells to reorganize their metabolism, increase ATP and NADH/NADPH production, and adapt the cell cycle to higher energy demands, likely through activation of replication checkpoints [37]; by doing so, the cell is able to survive despite limited proliferation. In KURAMOCHI cells, the decrease in the S-phase population after nine days of DEX exposure, without significant changes in other cell-cycle phases, suggests that the drug may limit entry into DNA replication, leading to reduced proliferation. The cell cycle appears to be slowed, yet remains balanced, and cells may utilize this time to reorganize their metabolism. However, in the context of the present study, these mechanisms remain speculative and warrant further investigation.

Dexamethasone also influenced apoptotic regulation. Consistent with previously reported findings in prostate cancer cells [15], prolonged DEX (100 nM) exposure in ovarian cancer cells also showed an association with a decrease in apoptotic cell numbers under low-serum conditions (Figure 4A). In SKOV3 cells, this effect was observed after short-term treatment, whereas in KURAMOCHI cells it occurred only after prolonged exposure and was preceded by a transient increase in apoptosis. Based on these observations, we hypothesize that DEX may exert effects consistent with a cytostatic-like response in ovarian cancer cells, potentially through differential modulation of the cell cycle, including G0/G1 arrest as well as slowing alteration of S-phase progression.

Although it was initially assumed that DEX mainly demonstrates pro-survival effects under stress conditions [15], our data indicate that DEX may also support cell survival in full-serum conditions (10% FBS). These findings are consistent with changes in the *BAX*/*BCL2* ratio (Figure 4B), particularly under full-serum conditions, suggesting that the effects of DEX on cell survival operate in a time- and context-dependent manner. Although tumor dormancy was not directly investigated in this study, the observed pro-survival effects of DEX may hypothetically contribute to dormant-like cellular states, which require further functional validation.

There is increasing preclinical evidence that DEX may induce resistance to cytotoxic drugs such as cisplatin and paclitaxel [38,39] and enhance the cancer cell properties associated with stemness [40]. However, the treatment efficacy of DEX supplementation in cytotoxic therapy against solid cancers remains to be tested in large randomized clinical trials. Despite this, some studies have examined the effects of omitting DEX in chemotherapy regimens, and some retrospective clinical data suggests that omitting GCs from the treatment regimen may sometimes achieve a better disease course [41,42,43].

Given that DEX can modulate the RAS [5,44,45] and that tissue RAS contributes to tumor development and aggressiveness [16,46], the study evaluated whether DEX modulates the expression of RAS receptors in a time- and context-dependent manner. Our results indicate that significant changes in angiotensin receptor expression occur primarily after prolonged exposure (Table 1), which may reflect the expected kinetics of glucocorticoid receptor–dependent transcriptional responses. In KURAMOCHI cells, the observed effects were strongly dependent on serum concentration, whereas in SKOV3 cells, they were largely independent of culture conditions. In KURAMOCHI cells, all four receptors were upregulated under serum-deprived conditions, although *AGTR2* did not show a significant change. Under low-serum conditions, cells are more susceptible to stress and may engage stress-adaptive signaling pathways, potentially including components of the local RAS. KURAMOCHI cells more closely replicate the morphology of serous ovarian cancer and are more sensitive to changes in the tumor microenvironment [47]. After nine days of DEX exposure in SKOV3 cells, all angiotensin receptors showed an upward trend, regardless of serum concentration in the medium. However, statistically significant changes were observed only for *AGTR1* and *LNPEP* under full-serum culture conditions.

In addition, elevated *AGTR1* expression was associated with poorer OS and DFS in ovarian cancer, whereas increased *LNPEP* correlated with reduced OS (Table 2). These observations suggest that the DEX-induced changes in angiotensin receptor-related gene expression observed after nine days of exposure may reflect transcriptional patterns associated with less favorable clinical outcomes in ovarian cancer.

Importantly, *LNPEP* (AT4/IRAP) may represent a potential component linking GC signaling with the RAS. In prostate cancer, only AT4/IRAP, the most abundant angiotensin receptor in the studied cell lines, decreased following DEX exposure, with the only significant change observed in DU-145 cells. In contrast, *LNPEP* expression was upregulated in KURAMOCHI cells, following DEX exposure under serum-deprived conditions. Our DepMap demonstrated a positive correlation between *LNPEP* and *NR3C1* expression in both ovarian and prostate cancer cell lines, which was further supported by analyses of patient-derived datasets using GEPIA. Similar expression patterns were also observed in our RT-qPCR experiments. Together, these findings suggest the existence of possible transcriptomic associations between *LNPEP* and *NR3C1* expression that may reflect coordinated responses to DEX exposure.

This observation is supported by large-scale transcriptomic meta-analyses of the extended RAS (extRAAS), based on human atherosclerotic tissue samples. The findings demonstrated that *LNPEP* and *NR3C1* cluster within the same co-expression module together with other receptor-encoding genes, including *AGTR1*. This receptor-associated module is distinct from enzymatic RAS components and shows inverse correlation with enzyme-enriched modules, indicating a higher-order organization of the RAS into transcriptionally coordinated functional units. Although these data originate from vascular tissue, this systems-level organization suggests that conserved regulatory relationships may exist within the extended RAS potentially extending beyond the cardiovascular context. In cancer cells, such coordination may contribute to integrated regulation of GCs and angiotensin signaling pathways [47]. However, in the context of the present study, this hypothesis remains speculative and warrants further investigation. Future studies involving GR inhibition and functional analyses of angiotensin receptors will be necessary to validate this proposed mechanism. If direct transcriptional regulation by GR is involved, ChIP-based approaches would also be required.

Previous research has found AT4/IRAP to modulate GLUT4 activity and insulin signaling, enabling cells to control glucose uptake and adapt their metabolism to environmental conditions [48]. The opposing effects of DEX on *LNPEP* according to tumor type appear to reflect their distinct energy metabolisms: ovarian cancer is predominantly glycolytic, whereas prostate cancer relies more on oxidative phosphorylation and lipid metabolism [33,49,50,51]. These different metabolic strategies may be associated with the observed differences in LNPEP regulation following DEX treatment, while the overall direction of cellular responses remained consistent across both tumor types. An analysis of TCGA data by Ma et al. [52] identified significantly lower *LNPEP* mRNA expression in ovarian cancer compared to adjacent normal tissues; however, higher *LNPEP* expression was associated with shorter OS, as well as poorer DFS and PFS. Based on these findings, we hypothesize that DEX may influence LNPEP-associated biological processes; however, the precise functional consequences of such modulation remain to be elucidated. Ma et al. [52] also report that in ovarian cancer, *LNPEP* co-expressed genes are primarily associated with immune-related pathways, including Th1, Th2, and Th17 cell differentiation and immunoregulatory interactions; *LNPEP* expression also correlates with immune cell infiltration in the tumor, suggesting that it may have a role in the immunological mechanisms in this region [52].

Interestingly, *LNPEP* is known to participate in the inflammatory response by activating the NF-κB pathway via Ang IV, and to prepare peptides for presentation to T lymphocytes by trimming in dendritic cell endosomes [53]. It is noteworthy that in the previous study, significant decreases in the expression of multiple NF-κB family members were noted in prostate cancer after nine days of DEX exposure under serum-deprived conditions; in addition, this decrease corresponded with the downregulation of *LNPEP* observed in a previous study [15]. These findings suggest that DEX exposure may be associated with changes in LNPEP expression linked to inflammatory- and immune-related pathways. The observed differences between ovarian and prostate cancer models may reflect tissue-specific modulation of molecular pathways by DEX although further mechanistic studies are required to confirm this. Although the direction of LNPEP expression changes differed between the analyzed cancer types, the observed transcriptomic patterns were associated with less favorable survival outcomes in both models.

Despite the biological relevance of the presented observations, several limitations should be considered. An important limitation of this study is the lack of protein-level and functional validation of the analyzed signaling components. Since *AGTR1*, *AGTR2*, *MAS1*, *LNPEP*, and *NR3C1* are functional signaling proteins, transcriptomic data alone may not fully reflect receptor abundance, activity, or downstream pathway activation. This limitation is particularly relevant for genes with low expression levels in both DepMap datasets and our qPCR analyses, where linearity of the measured signal cannot always be assumed. Accordingly, the present findings should be interpreted as transcriptomic and association-based observations, and will require further validation at the protein and functional levels in future studies. An additional limitation of the study is that the survival analyses were based on univariate Kaplan–Meier stratification without adjustment for clinically relevant covariates, including age, tumor stage, grade, treatment status, or molecular subtype. Therefore, the presented associations should not be interpreted as evidence of independent prognostic value. Furthermore, the analyses were conducted using publicly available datasets originating from multiple studies and transcriptomic platforms, which may introduce cohort heterogeneity and potential batch-related effects. Although cohort selection was restricted according to tumor type, residual biological and technical variability cannot be excluded.

## 5. Conclusions

In summary, long-term exposure (nine days) of ovarian and prostate cancer cells to DEX induces adaptive changes which allow the cell to survive unfavorable environmental conditions, including serum deprivation. Dexamethasone may promote a survival-supportive state in cancer cells in a tissue-specific manner, associated with slowing or arresting the cell cycle, reprogramming the metabolism, inhibiting apoptosis, and modulating immune-related signaling in the tumor context. Importantly, our findings demonstrate that DEX modulates the expression of angiotensin receptors in both ovarian and prostate cancer cells in a time- and serum-dependent manner, potentially affecting both OS and DFS. Taken together, these findings suggest a possible role of the local RAS in DEX-induced cellular adaptation. In addition, our findings revealed a consistent correlation between *NR3C1* and *LNPEP* in *in vitro* ovarian and androgen-insensitive prostate cancer models and patient-derived datasets, irrespective of tumor origin. Although the functional significance of this observation requires further investigation, these results suggest the presence of transcriptomic associations between glucocorticoid signaling- and RAS-related genes in the analyzed cancer models.

## Figures and Tables

**Table 1:** Median fold changes in gene expression (*AGTR1*—angiotensin II receptor type 1, *AGTR2*—angiotensin II receptor type 2, *LNPEP*—insulin-regulated aminopeptidase, *MAS1*—*MAS1* proto-oncogene, G protein-coupled receptor, and *NR3C1*—glucocorticoid receptor) following DEX treatment in ovarian and prostate cancer cells. Median values are presented to highlight central tendencies and minimize the influence of outliers (*n* ≥ 3). The arrows indicate the direction of change: ↑ significant increase, ↓ significant decrease (one-way ANOVA with Tukey’s test or Kruskal–Wallis test with Dunn’s multiple comparisons: *p*  <  0.05).

**Gene**	**Ovarian Cancer**							
	**Kuramochi**				**SKOV3**			
	**100 nM DEX;** **3 Days**		**100 nM DEX;** **9 Days**		**100 nM DEX;** **3 Days**		**100 nM DEX;** **9 Days**	
	**5% FBS**	**10% FBS**	**5% FBS**	**10% FBS**	**5% FBS**	**10% FBS**	**5% FBS**	**10% FBS**
*AGTR1* (AT1)	0.96	0.92	1.68 ↑	0.64	0.93	0.81	1.21	2.68 ↑
*AGTR2* (AT2)	0.91	1.24	1.39	0.55	0.80	0.45	2.09	2.06
*LNPEP* (AT4)	1.15	1.09	1.82 ↑	0.61	0.85	0.60	1.16	1.82 ↑
*MAS1*	1.09	1.58	1.75 ↑	0.74	0.86	0.49	1.25	2.78
*NR3C1* (GR)	1.14	0.88	1.31 ↑	0.68 ↓	0.90	0.82	0.98	1.10
**Gene**	**Prostate Cancer**							
	**DU-145**				**PC3**			
	**100 nM DEX;** **9 Days**		**10 nM DEX;** **9 Days**		**100 nM DEX;** **9 Days**		**10 nM DEX;** **9 Days**	
	**5% FBS**				**5% FBS**			
*AGTR1* (AT1)	0.82		1.04		1.34		1.19	
*AGTR2* (AT2)	1.39		1.17		0.86		0.52	
*LNPEP* (AT4)	0.37 ↓		0.37 ↓		0.48		0.96	
*MAS1*	1.22		2.06		1.09		1.52	
*NR3C1* (GR)	0.69		0.64		0.65		0.91	

**Table 2:** Gene expression and survival in ovarian and prostate cancer. OS, overall survival; DFS, disease-free survival; PFS, progression-free survival. For each analysis, the number of patients, *p*-value, *q*-value (false discovery rate, FDR), and hazard ratio (HR) with 95% confidence interval (CI) are presented. Results with *q* < 0.05 remained significant after correction for multiple testing. NE (not estimable) indicates that the hazard ratio could not be reliably estimated due to the absence of events in one comparison group.

Gene	Survival Type	Ovarian				Prostate			
		Number of Patients	*p*-Value	*q*-Value	HR (95% CI)	Number of Patients	*p*-Value	*q*-Value	HR (95% CI)
*AGTR1*	OS	301	7.975 × 10^−3^	0.0160	1.499 (1.113–2.020)	501	0.356	0.536	1.602 (0.601–4.269)
	DFS	197	6.707 × 10^−3^	0.0160	1.574 (1.120–2.211)	400	0.402	0.536	0.814 (0.502–1.319)
	PFS	150	0.104	0.118	1.373 (0.933–2.018)	251	0.357	0.536	0.774 (0.449–1.333)
*AGTR2*	OS	300	0.558	0.558	0.920 (0.693–1.221)	329	0.620	0.897	NE
	DFS	213	9.890 × 10^−6^	1.978 × 10^−5^	0.496 (0.361–0.681)	225	0.897	0.897	1.098 (0.279–4.314)
	PFS	-	-	-	-	-	-	-	-
*LNPEP*	OS	511	2.869 × 10^−3^	5.738 × 10^−3^	1.404 (1.117–1.763)	491	0.0598	0.119	0.170 (0.039–0.746)
	DFS	423	0.365	0.365	1.108 (0.886–1.385)	416	0.0365	0.119	0.590 (0.357–0.975)
	PFS	-	-	-	-	239	0.0895	0.119	0.588 (0.321–1.078)
*MAS1*	OS	507	0.0161	0.0322	1.319 (1.047–1.662)	548	0.438	0.438	0.545 (0.138–2.157)
	DFS	427	0.602	0.602	1.060 (0.850–1.323)	514	0.0837	0.167	0.662 (0.435–1.030)
	PFS	-	-	-	-	-			-
*NR3C1*	OS	301	0.0390	0.0779	1.358 (1.006–1.833)	503	0.0345	0.0460	NE
	DFS	199	0.0220	0.0779	1.477 (1.043–2.090)	415	6.965 × 10^−4^	2.786 × 10^−3^	0.419 (0.258–0.682)
	PFS	149	0.0630	0.0840	1.447 (0.972–2.153)	250	2.740 × 10^−3^	5.481 × 10^−3^	0.430 (0.252–0.733)

## Data Availability

The raw data or unpublished data that support the findings of this study are available upon request from the corresponding author.

## Supplementary Materials

The following supporting information can be downloaded at https://www.mdpi.com/article/10.3390/cancers18121998/s1: Table S1: Details of primer sequences, annealing temperature, amplicon length, and amplification location for target genes. Table S2: Stability of reference gene Ct values across ovarian and prostate cancer cell lines. Values are based on *n* = 120 measurements per gene. Inter-sample Ct range was calculated as the difference between the highest and lowest mean Ct values across cell lines (max − min). Table S3: RT-qPCR assay validation parameters for receptor genes determined from serial dilutions of pooled cDNA samples. The table summarizes Ct ranges, standard curve slopes, coefficients of determination (R^2^), and overall assay performance for reference and target genes. Figure S1: Melting curve analyses for *AGTR1* (A), *AGTR2* (B), *MAS1* (C), *LNPEP* (D), *NR3C1* (E), *BCL2* (F), *BAX* (G), *H3F3A* (H), *RPLP0* (I) obtained using serial dilutions of pooled cDNA samples (1:1, 1:5, 1:25, and 1:125). Pooled cDNA was generated from all cell lines and experimental conditions included in the study. Figure S2: Kaplan–Meier survival analysis for prostate cancer patients stratified by gene expression (*AGTR1*, *AGTR2*, *LNPEP*, *MAS1*, and *NR3C1*). Kaplan–Meier curves illustrating overall survival (OS), disease-free survival (DFS), and progression-free survival (PFS) in prostate cancer patients, stratified according to gene expression levels. Patients were divided into groups based on the lowest (Q1) and highest (Q4) quartiles of mRNA expression. Expression values were defined as z-scores relative to all samples (log RNA-seq V2 RSEM or log microarray data, depending on dataset availability). Analyses were performed using the cBioPortal platform with default parameters. The prostate cancer cohort was restricted to cases classified as prostate adenocarcinoma (*n* = 13,296). Survival differences between groups were evaluated using the log-rank test, with corresponding *p*-values indicated on the plots. Figure S3: Kaplan–Meier survival analysis for ovarian cancer patients stratified by gene expression (*AGTR1*, *AGTR2*, *LNPEP*, *MAS1*, and *NR3C1*). Kaplan–Meier curves illustrating overall survival (OS), disease-free survival (DFS), and progression-free survival (PFS) in ovarian cancer patients, stratified according to gene expression levels. Patients were divided into groups based on the lowest (Q1) and highest (Q4) quartiles of mRNA expression. Expression values were defined as z-scores relative to all samples (log RNA-seq V2 RSEM or log microarray data, depending on dataset availability). Analyses were performed using the cBioPortal platform with default parameters. The ovarian cancer cohort was filtered to include serous ovarian cancer, high-grade serous ovarian cancer, and ovarian cancer cases (*n* = 2562). Survival differences between groups were evaluated using the log-rank test, with corresponding *p*-values indicated on the plots. Figure S4: CRISPR knockout effects on cell viability for *AGTR1*, *AGTR2*, *LNPEP*, *MAS1*, and *NR3C1* in KURAMOCHI (A), SKOV3 (B), DU-145 (C), and PC3 (D) cells, based on DepMap. Figure S5: Correlation analysis of gene expression in prostate cancer. Scatter plots illustrating pairwise correlations between the indicated genes in prostate adenocarcinoma samples from the TCGA-PRAD cohort. Analyses were performed using the GEPIA platform, based on data from The Cancer Genome Atlas. Pearson correlation coefficients (R) and corresponding *p*-values are shown on each plot. Non-log-transformed expression values were used for statistical calculations, while log-scale transformation was applied for data visualization. Figure S6: Correlation analysis of gene expression in ovarian cancer. Scatter plots illustrating pairwise correlations between the indicated genes in ovarian cancer samples from the TCGA-OV cohort. Analyses were performed using the GEPIA platform, based on data from The Cancer Genome Atlas. Pearson correlation coefficients (R) and corresponding *p*-values are shown on each plot. Non-log-transformed expression values were used for statistical calculations, while log-scale transformation was applied for data visualization.

## Abbreviations

The following abbreviations are used in this manuscript:

DEX	Dexamethasone
GC	Glucocorticoid
RAS	Renin–Angiotensin System
GR	glucocorticoid receptor
OCCC	clear cell ovarian carcinoma
HGSOC	high-grade serous ovarian carcinoma
STR	short tandem repeat
ATCC	American Type Culture Collection
ECACC	European Collection of Authenticated Cell Cultures
FBS	fetal bovine serum
PBS	phosphate-buffered saline
qPCR	Quantitative PCR
GEPIA	Gene Expression Profiling Interactive Analysis
OS	overall survival
DFS	disease-free survival
PFS	progression-free survival
SD	standard deviation
IQR	interquartile range
*AGTR1*	angiotensin II receptor type 1
*AGTR2*	angiotensin II receptor type 2
*LNPEP*	insulin-regulated aminopeptidase
*MAS1*	Proto-oncogene Mas
*NR3C1*	glucocorticoid receptor
extRAAS	extended RAS
MRD	minimal residual disease
