# Supplementary material for: Dexamethasone as a Modulator of Renin–Angiotensin System Receptor Expression in Prostate and Ovarian Cancer Cells Under Standard and Low-Serum Conditions
Source: Cancers (Basel). 2026 Jun 19;18(12):1998. doi: 10.3390/cancers18121998 (PMC13297035; doi:10.3390/cancers18121998)
Supplement: Supplementary file 1 [file cancers-18-01998-s001.zip › cancers-4319036-supplementary.pdf]

Supplementary Table S1. Details of primer sequences, annealing temperature, amplicon length, and amplification location for target genes.

GENE SYMBOL	ANNEALING TEMPERATURE [°C]	SEQUENCE ACCESSION NUMBER	PRIMER SEQUENCES (5`-3`)	LOCATION OF AMPLICON [BP]	AMPLICON LENGTH [BP]	IN SILICO SPECIFICITY SCREEN - BLAST	PRIMER LOCATION	TARGETED SPLICE VARIANTS
H3F3A	65	NM_002107.7	F: AGGACTTTAAAACAGATCTGCGCTTCCAGAG R: ACCAGATAGGCCTCACTTGCCTCCTGC	349 - 424	76	Specific	Exon 3 - exon 4 junction	transcript variant 1 (NM_002107.7) transcript variant 2 (NM_001379043.1) transcript variant 3 (NM_001379045.1) transcript variant 4 (NM_001379046.1) transcript variant 5 (NM_001379047.1)
RPLP0	65	NM_001002.4	F: ACGGATTACACCTTCCCACTTGCTGAAAAGGTC R: AGCCACAAAGGCAGATGGATCAGCCAAG	840-908	69	Specific	Exon 7 - exon 8 junction	transcript variant 2 (NM_053275.4)
AGTR1	59	NM_000685.5	F: GTGGCTTTGCTTTGTCTTGT R: GAACATTCCTCTGCAGCACT	1486-1636	151	Specific	Exon 3	transcript variant 1 (NM_000685.5) transcript variant 2 (NM_009585.4) transcript variant 3 (NM_004835.5) transcript variant 4 (NM_031850.4) transcript variant 5 (NM_032049.4) transcript variant 6 (NM_001382736.1) transcript variant 7 (NM_001382737.1)
AGTR2	59	NM_000686.5	F: GTTCCCCTTGTTTGGTGTAT R: CATCTTCAGGACTTGGTCAC	658-939	282	Specific	Exon 3	transcript variant 1 (NM_000686.5) transcript variant 2 (NM_001385624.1)
MAS1	59	NM_002377.4	F: CTACATCACCCACCTGTCTATC R: GGCCAGAAGAAAGCTCATAATC	593-690	98	Specific	Exon 3	transcript variant 1 (NM_002377.4) transcript variant 2 (NM_001366704.2)
NR3C1	60	NM_000176.3	F: ACAGCATCCCTTTCTCAACAG R: AGATCCTTGGCACCTATTCCAAT	1501-1599	99	Specific	Exon 2	transcript variant 1 (NM_000176.3) transcript variant 2 (NM_001018074.1) transcript variant 3 (NM_001018075.1) transcript variant 4 (NM_001018076.2) transcript variant 5 (NM_001018077.1) transcript variant 6 (NM_001020825.2)

								transcript variant 7 (NM_001024094.2) transcript variant 8 (NM_001204265.2) transcript variant 9 (NM_001364180.2) transcript variant 10 (NM_001364181.2) transcript variant 11 (NM_001364182.1) transcript variant 12 (NM_001364183.2) transcript variant 13 (NM_001364184.2) transcript variant 14 (NM_001364185.1)
LNPEP	58	NM_005575.3	F: TGGTGGCTATTCCTGACTTTG R: TCTATCCGCCATTGAAGAAGTG	1332-1441	110	Specific	Exon 6	transcript variant 1 (NM_005575.3) transcript variant 2 (NM_175920.4)
BCL2	56	NM_000633.3	F: TTGGCCCCCGTTGCTTTTCCTC R: TCCCACTCGTAGCCCCTCTGCGAC	853 - 974	122	Specific	Exon 2	transcript variant beta (NM_000657.3) transcript variant 3 (NM_001438935.1)
BAX	56	NM_001291428.2	F: AGAGGTCTTTTTCCGAGTGGCAG C R: TTCTGATCAGTTCCGGCACCTTG	321 - 457	137	Specific	Exon 4 - exon 5 junction	transcript variant alpha (NM_138761.4) transcript variant beta (NM_004324.4) transcript variant gamma (NM_001291429.2) transcript variant delta (NM_138763.4) transcript variant zeta (NM_001291431.2) transcript variant lambda (NM_001291430.2) transcript variant sigma (NM_138764.5)

Supplementary Table S2: Stability of reference gene Ct values across ovarian and prostate cancer cell lines. Values are based on n = 120 measurements per gene. Inter-sample Ct range was calculated as the difference between the highest and lowest mean Ct values across cell lines (max − min).

**Gene**	***H3F3A***			
**Cell lines**	**Ct mean**	**SD**	**Min**	**Max**
KURAMOCHI	18.41	0.31	18.41	19.45
SKOV3	19.25	0.40	18.80	20.19
DU-145	19.71	0.49	19.01	20.76
PC3	19.85	0.33	19.12	20.64
Mean Ct ± SD across cell lines	19.47 ± 0.54			
Inter-sample Ct range	0.96			
**Gene**	***RPLP0***			
**Cell lines**	**Ct mean**	**SD**	**Min**	**Max**
KURAMOCHI	17.43	0.27	17.03	18.06
SKOV3	17.87	0.26	17.08	18.18
DU-145	16.89	0.22	16.48	17.29
PC3	17.31	0.39	16.11	17.93
Mean Ct ± SD across cell lines	17.34 ± 0.46			
Inter-sample Ct range	0.98			

Supplementary Table S3: RT-qPCR assay validation parameters for receptor genes determined from serial dilutions of pooled cDNA samples. The table summarizes Ct ranges, standard curve slopes, coefficients of determination (R^2), and overall assay performance for reference and target genes.

Gen	Ct min	Ct max	slope	R^2	Assessment
H3F3A	18.58	25.20	-3.28	0.998	stable reference
RPLP0	17.74	23.48	-3.10	0.996	stable reference
AGTR1	23.82	29.80	-3.08	0.992	acceptable performance
AGTR2	24.38	29.17	-2.25	0.999	compressed curve bias
MAS1	24.20	29.04	-2.23	0.998	compressed curve bias
LNPEP	23.41	29.55	-3.11	0.998	good performance
NR3C1	22.15	28.94	-3.35	0.999	good performance
BAX	22.59	27.94	-3.09	0.999	acceptable performance
BCL2	24.69	30.07	-3.10	0.992	good performance

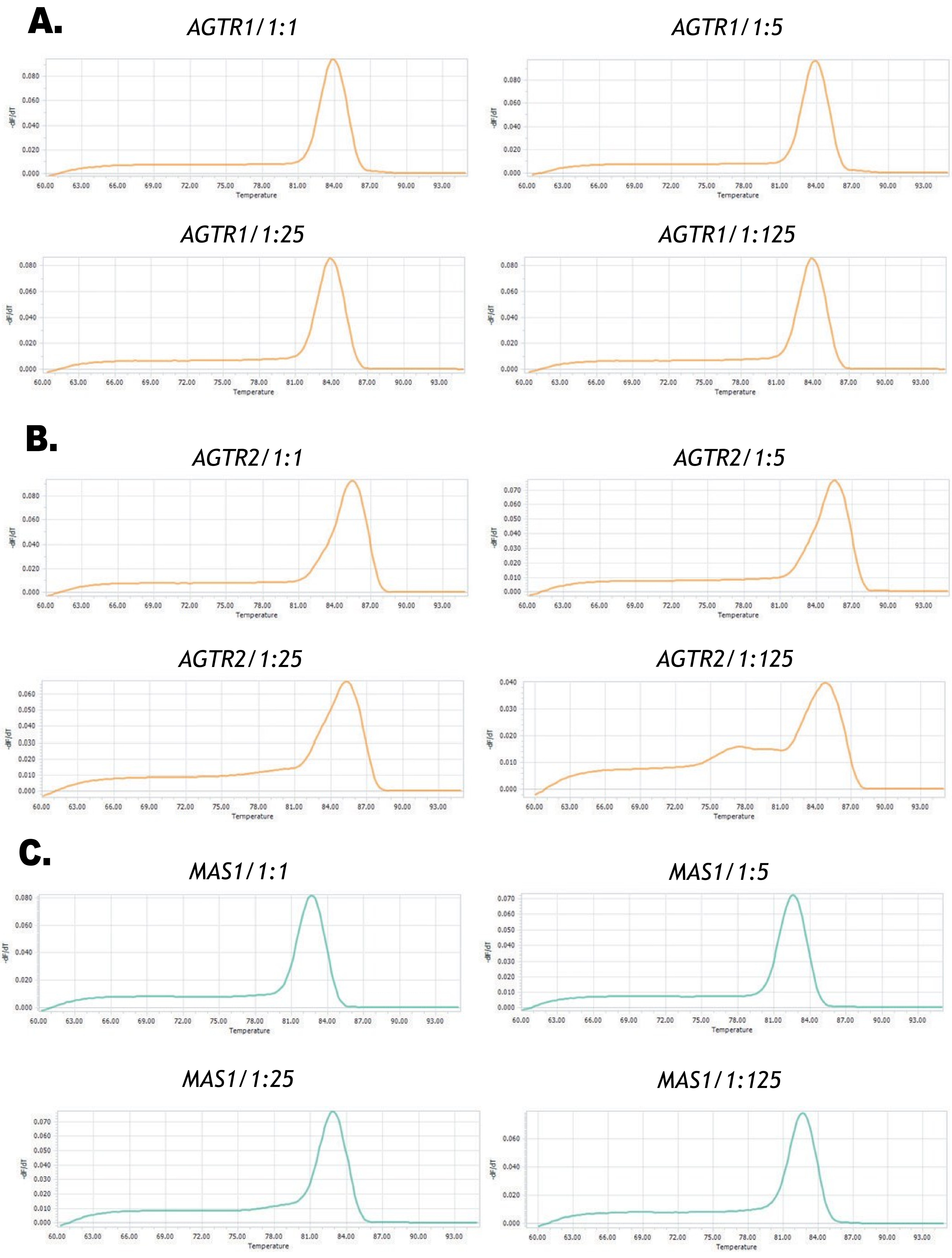
A.
AGTR1/1:1
AGTR1/1:5
AGTR1/1:25
AGTR1/1:125
B.
AGTR2/1:1
AGTR2/1:5
AGTR2/1:25
AGTR2/1:125
C.
MAS1/1:1
MAS1/1:5
MAS1/1:25
MAS1/1:125
-dF/dT
Temperature

D.

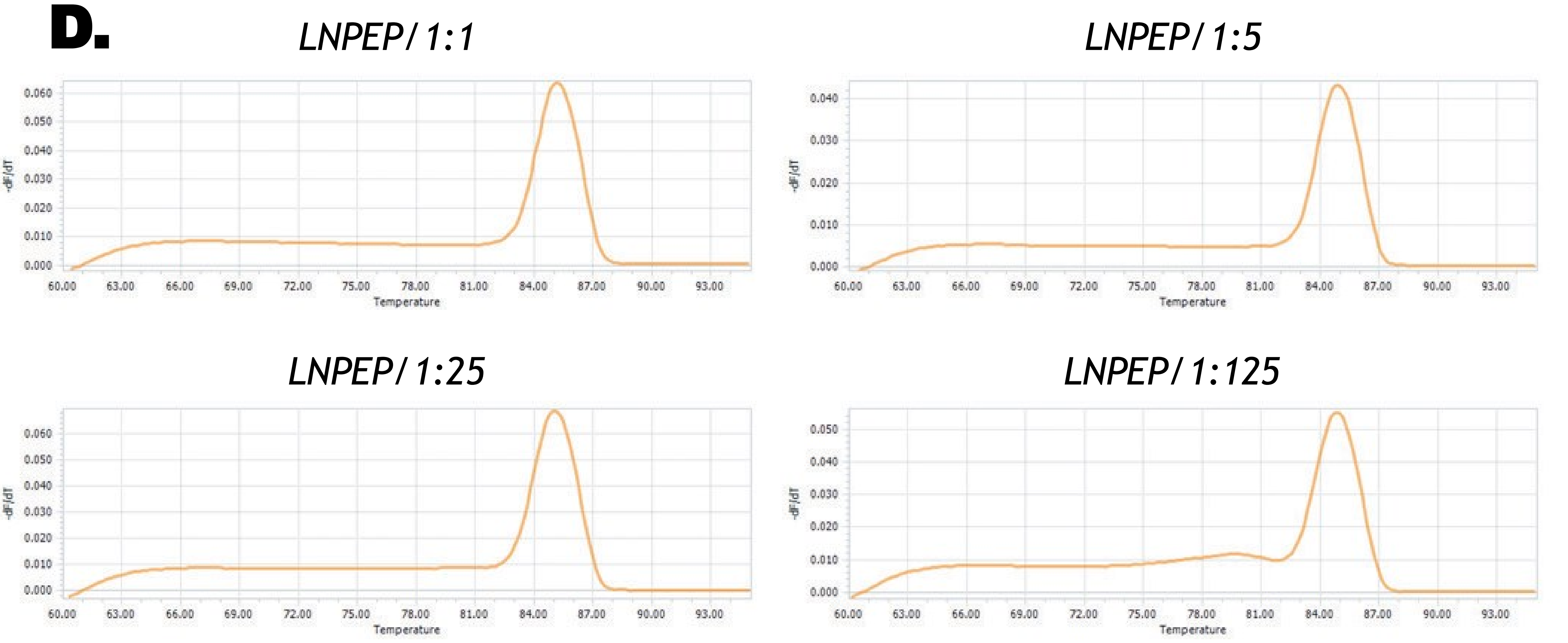

LNPEP/ 1:1
LNPEP/ 1:5
LNPEP/ 1:25
LNPEP/ 1:125
-dF/dT
Temperature

E.

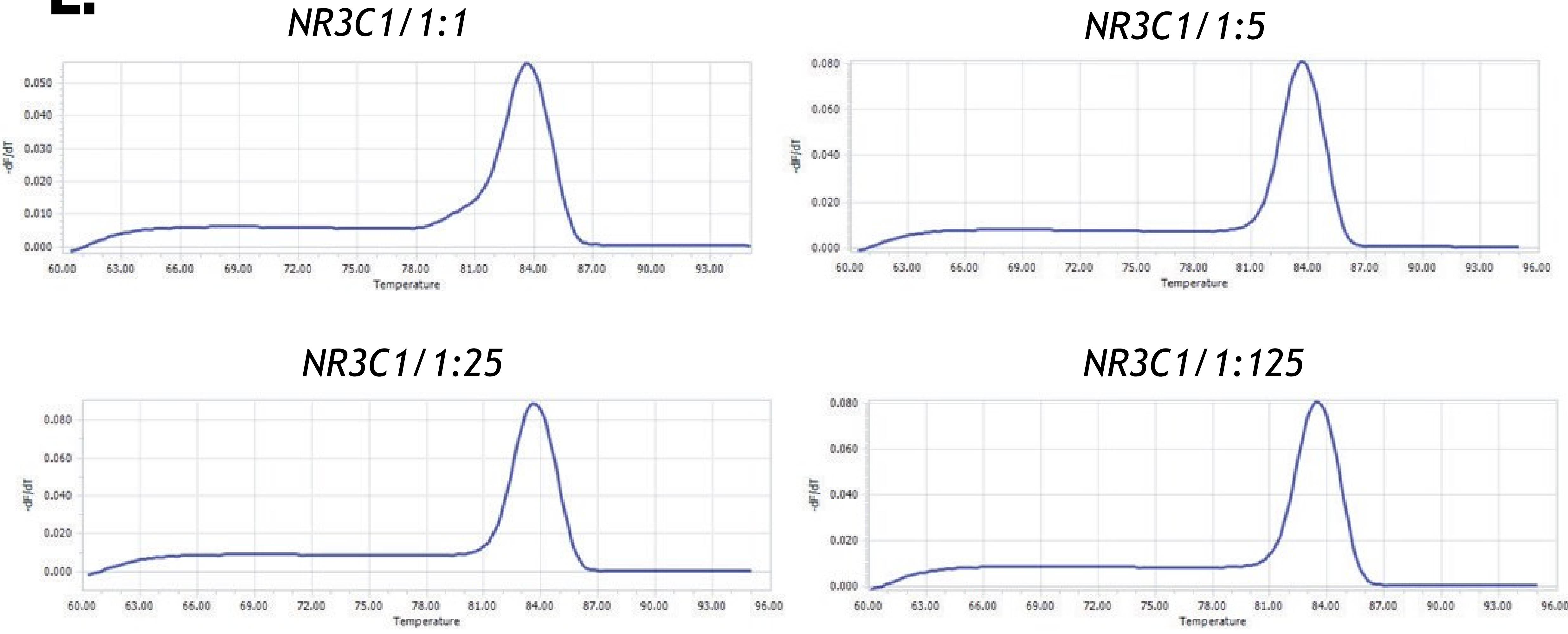

NR3C1/ 1:1
NR3C1/ 1:5
NR3C1/ 1:25
NR3C1/ 1:125
-dF/dT
Temperature

F.

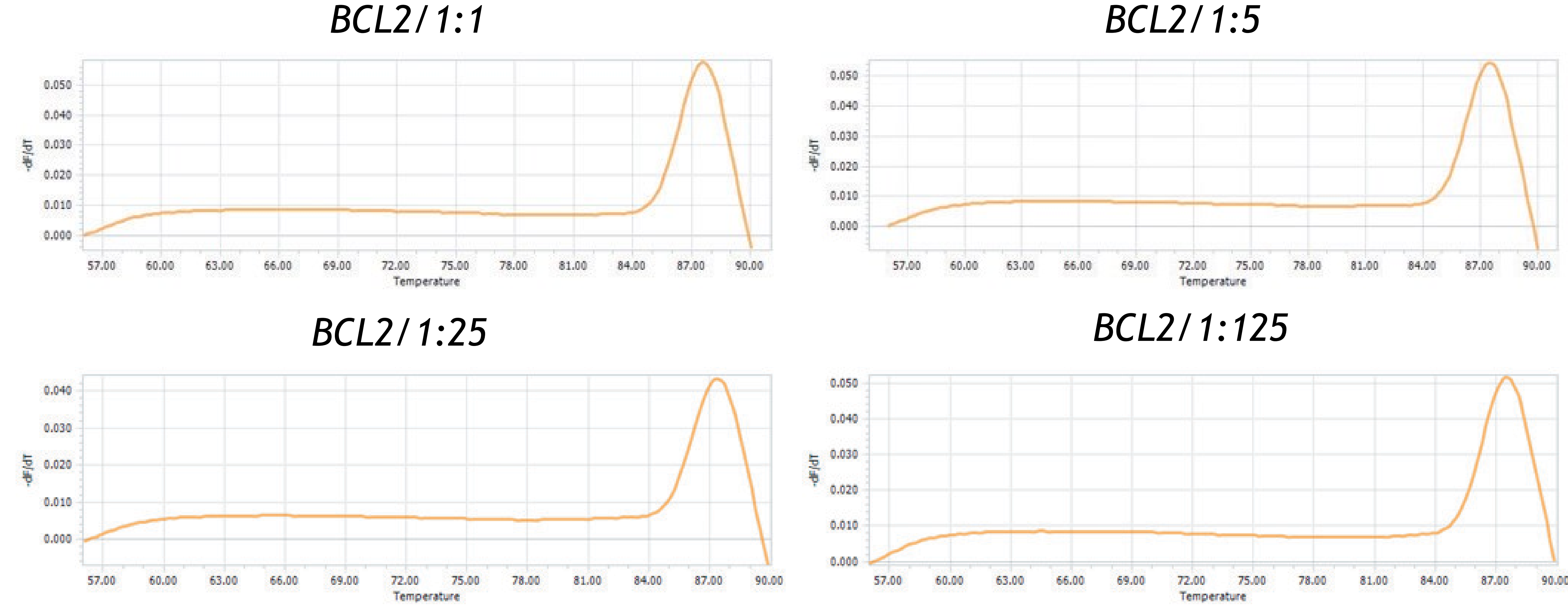

BCL2/ 1:1
BCL2/ 1:5
BCL2/ 1:25
BCL2/ 1:125
-dF/dT
Temperature

G.

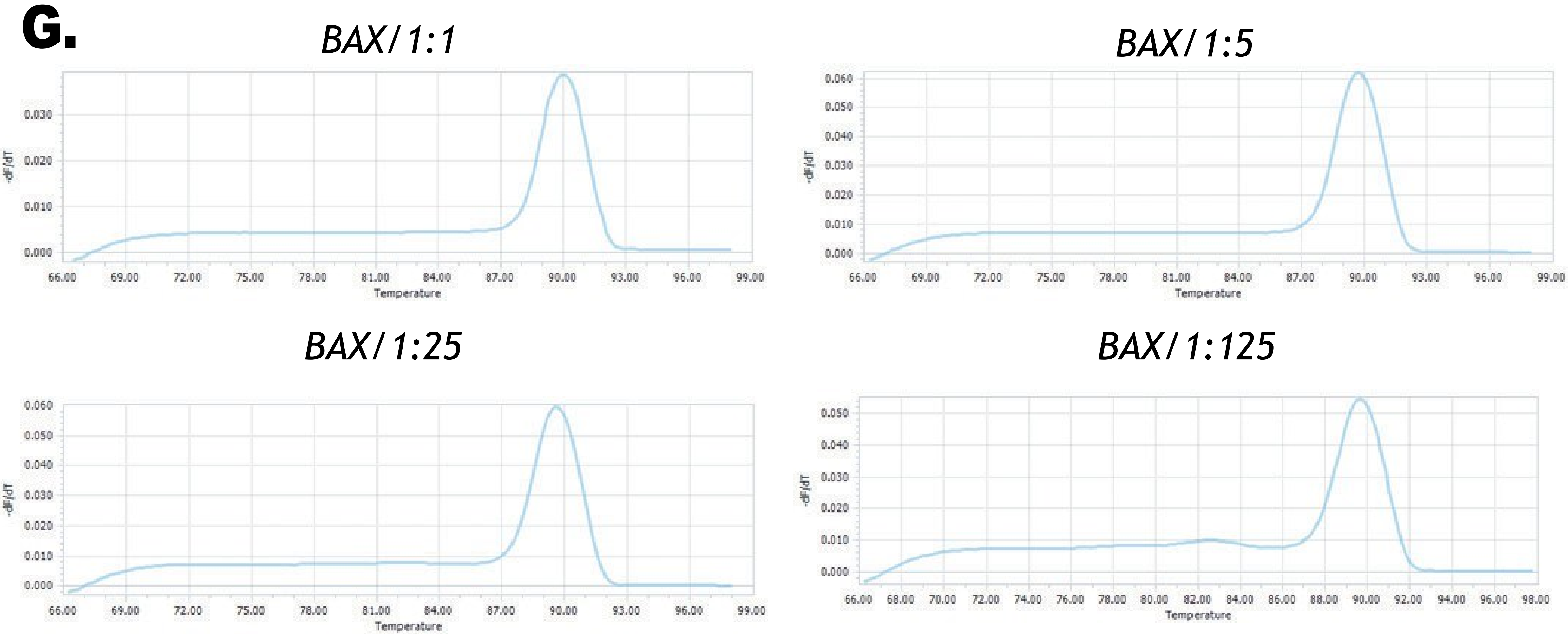

H.

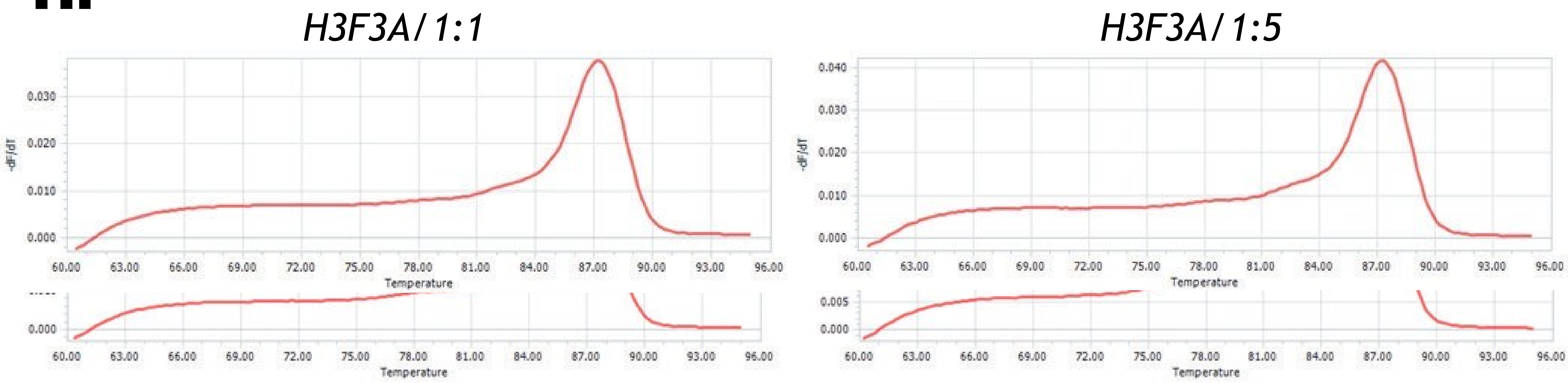

I.

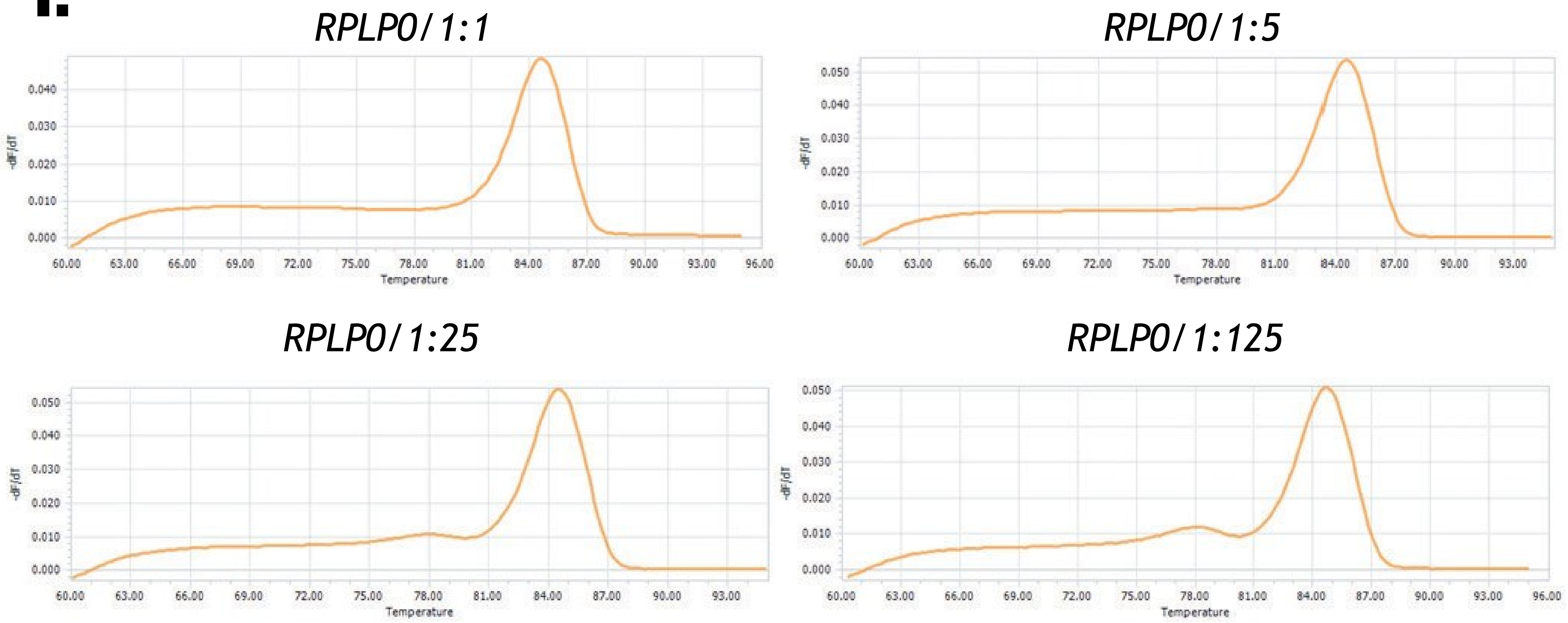

Supplementary Figure S1. Melting curve analyses for *AGTR1 (A), AGTR2 (B), MAS1 (C), LNPEP (D), NR3C1 (E), BCL2 (F), BAX (G), H3F3A (H), RPLP0 (I)* obtained using serial dilutions of pooled cDNA samples (1:1, 1:5, 1:25, and 1:125). Pooled cDNA was generated from all cell lines and experimental conditions included in the study.

PROSTATE CANCER

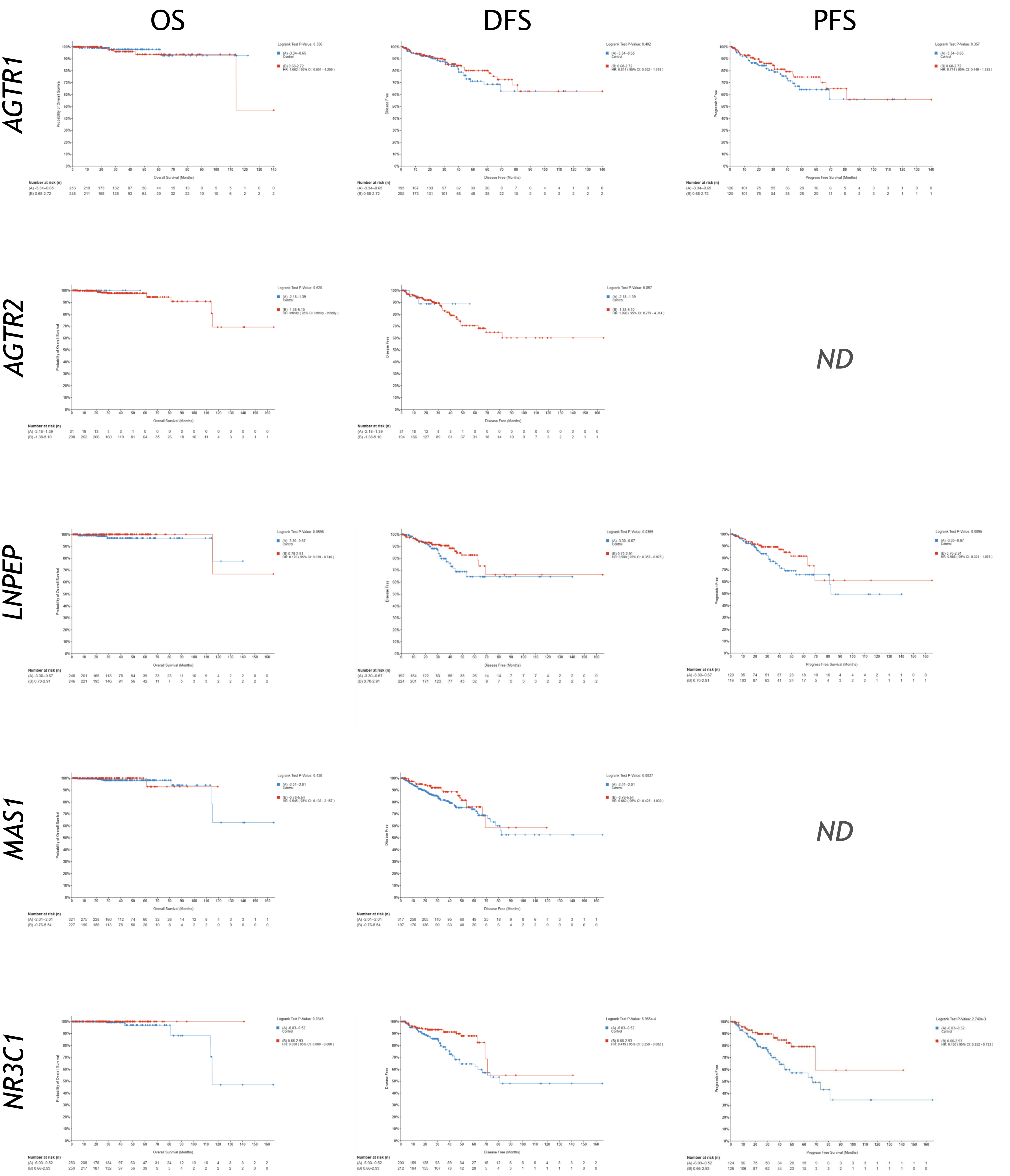

Supplementary Figure S2. Kaplan–Meier survival analysis for prostate cancer patients stratified by gene expression *(AGTR1, AGTR2, LNPEP, MAS1, and NR3C1)*. Kaplan–Meier curves illustrating overall survival (OS), disease-free survival (DFS), and progression-free survival (PFS) in prostate cancer patients, stratified according to gene expression levels. Patients were divided into groups based on the lowest (Q1) and highest (Q4) quartiles of mRNA expression. Expression values were defined as z-scores relative to all samples (log RNA-seq V2 RSEM or log microarray data, depending on dataset availability). Analyses were performed using the cBioPortal platform with default parameters. The prostate cancer cohort was restricted to cases classified as prostate adenocarcinoma (n = 13,296). Survival differences between groups were evaluated using the log-rank test, with corresponding p-values indicated on the plots.

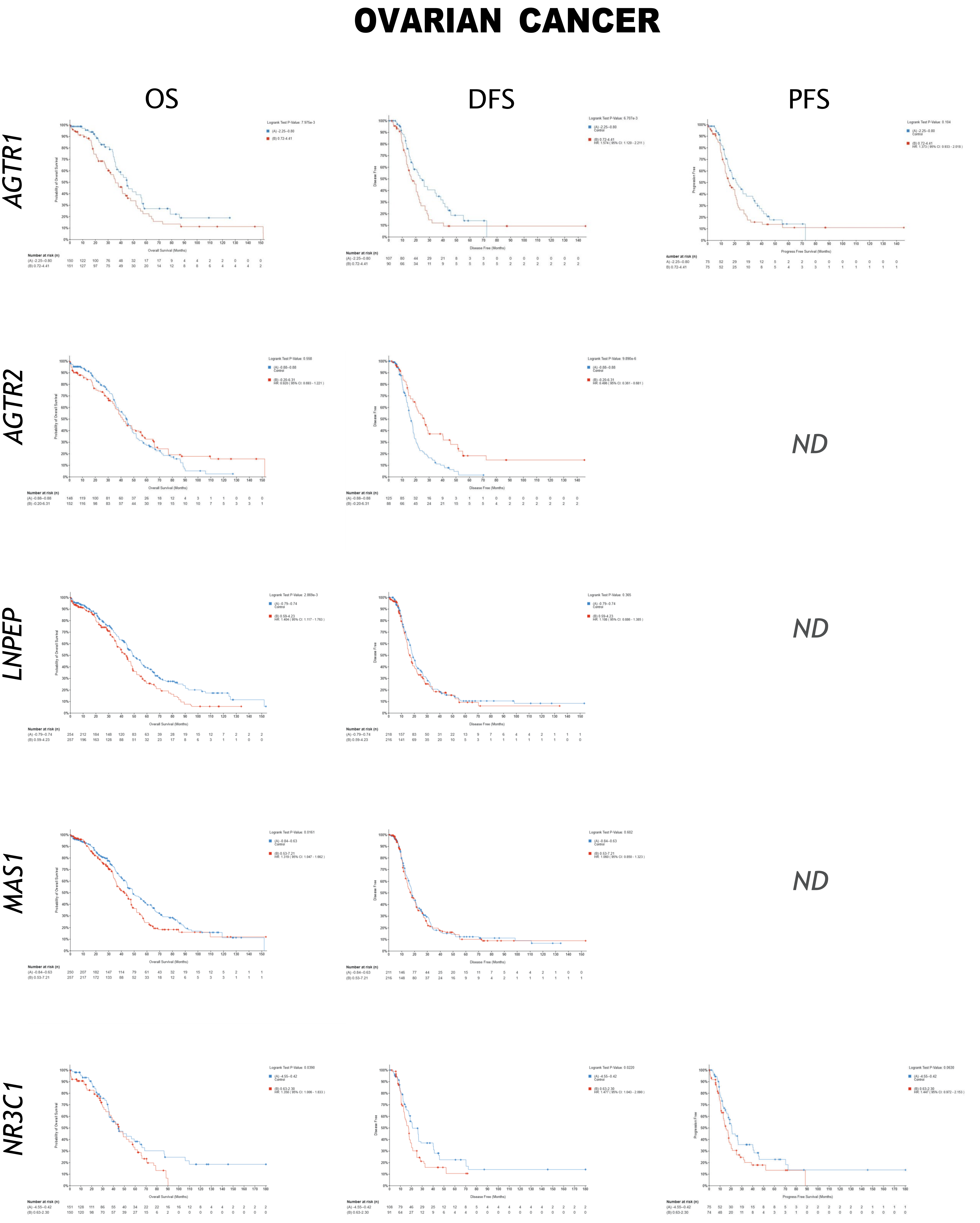

Supplementary Figure S3. Kaplan–Meier survival analysis for ovarian cancer patients stratified by gene expression *(AGTR1, AGTR2, LNPEP, MAS1, and NR3C1).* Kaplan–Meier curves illustrating overall survival (OS), disease-free survival (DFS), and progression-free survival (PFS) in ovarian cancer patients, stratified according to gene expression levels. Patients were divided into groups based on the lowest (Q1) and highest (Q4) quartiles of mRNA expression. Expression values were defined as z-scores relative to all samples (log RNA-seq V2 RSEM or log microarray data, depending on dataset availability). Analyses were performed using the cBioPortal platform with default parameters. The ovarian cancer cohort was filtered to include serous ovarian cancer, high-grade serous ovarian cancer, and ovarian cancer cases (n = 2562). Survival differences between groups were evaluated using the log-rank test, with corresponding p-values indicated on the plots.

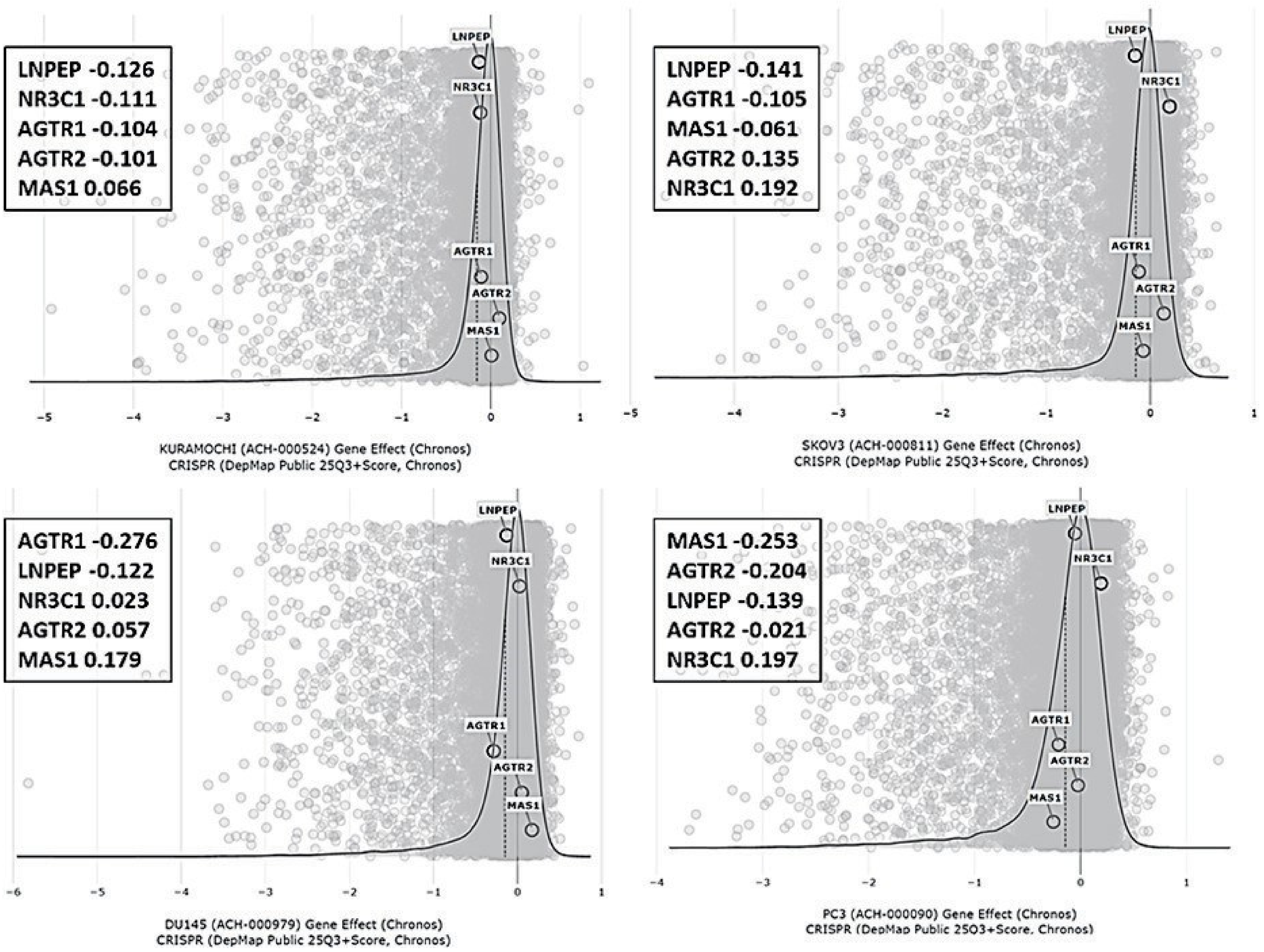

Supplementary Figure S4. CRISPR knockout effects on cell viability for *AGTR1, AGTR2, LNPEP, MAS1*, and *NR3C1* in KURAMOCHI (A), SKOV3 (B), DU-145 (C), and PC3 (D) cells, based on DepMap.

PROSTATE CANCER

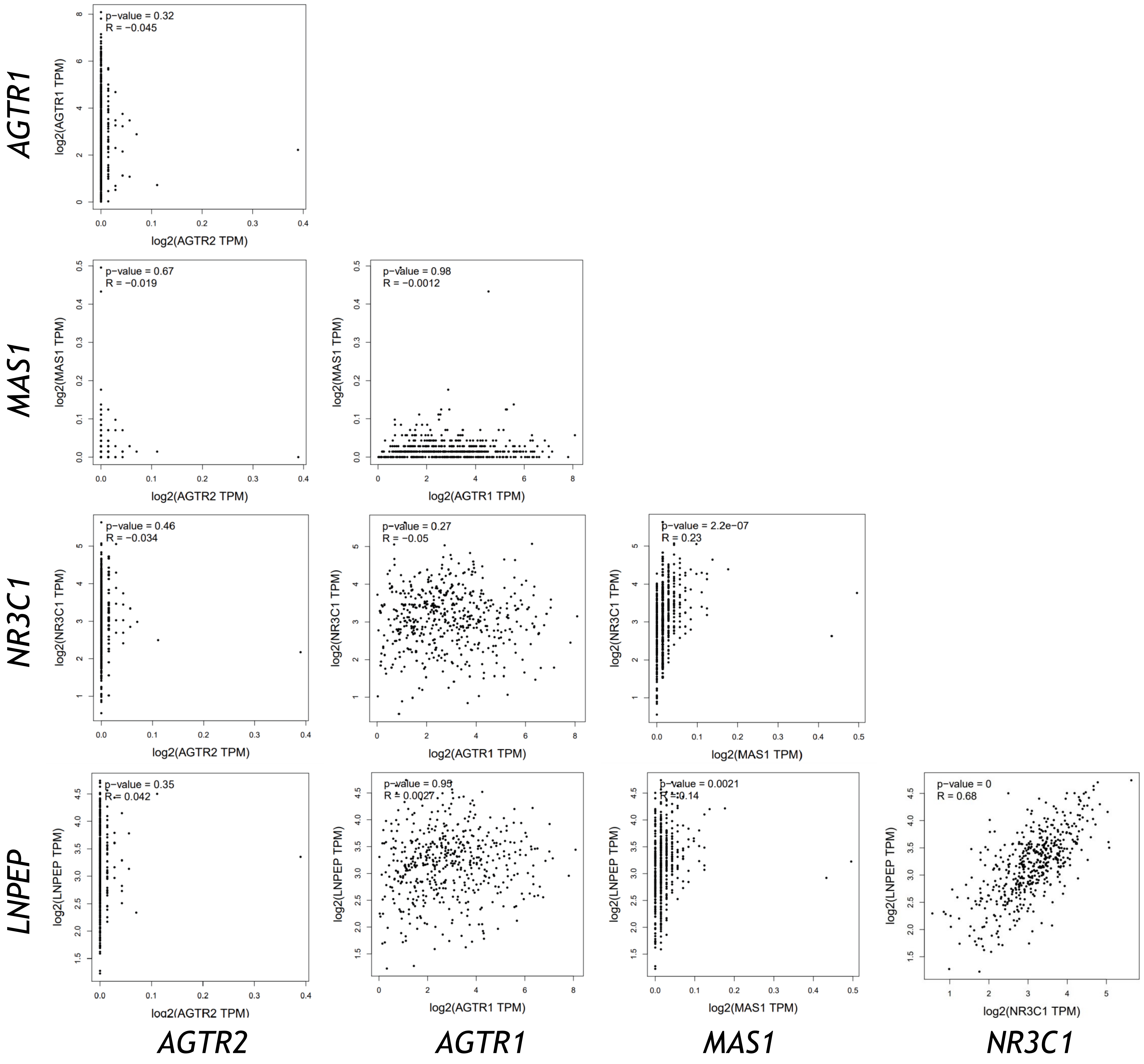

Supplementary Figure S5. Correlation analysis of gene expression in prostate cancer. Scatter plots illustrating pairwise correlations between the indicated genes in prostate adenocarcinoma samples from the TCGA-PRAD cohort. Analyses were performed using the GEPIA platform, based on data from The Cancer Genome Atlas. Pearson correlation coefficients (R) and corresponding p-values are shown on each plot. Non-log-transformed expression values were used for statistical calculations, while log-scale transformation was applied for data visualization.

OVARIAN CANCER

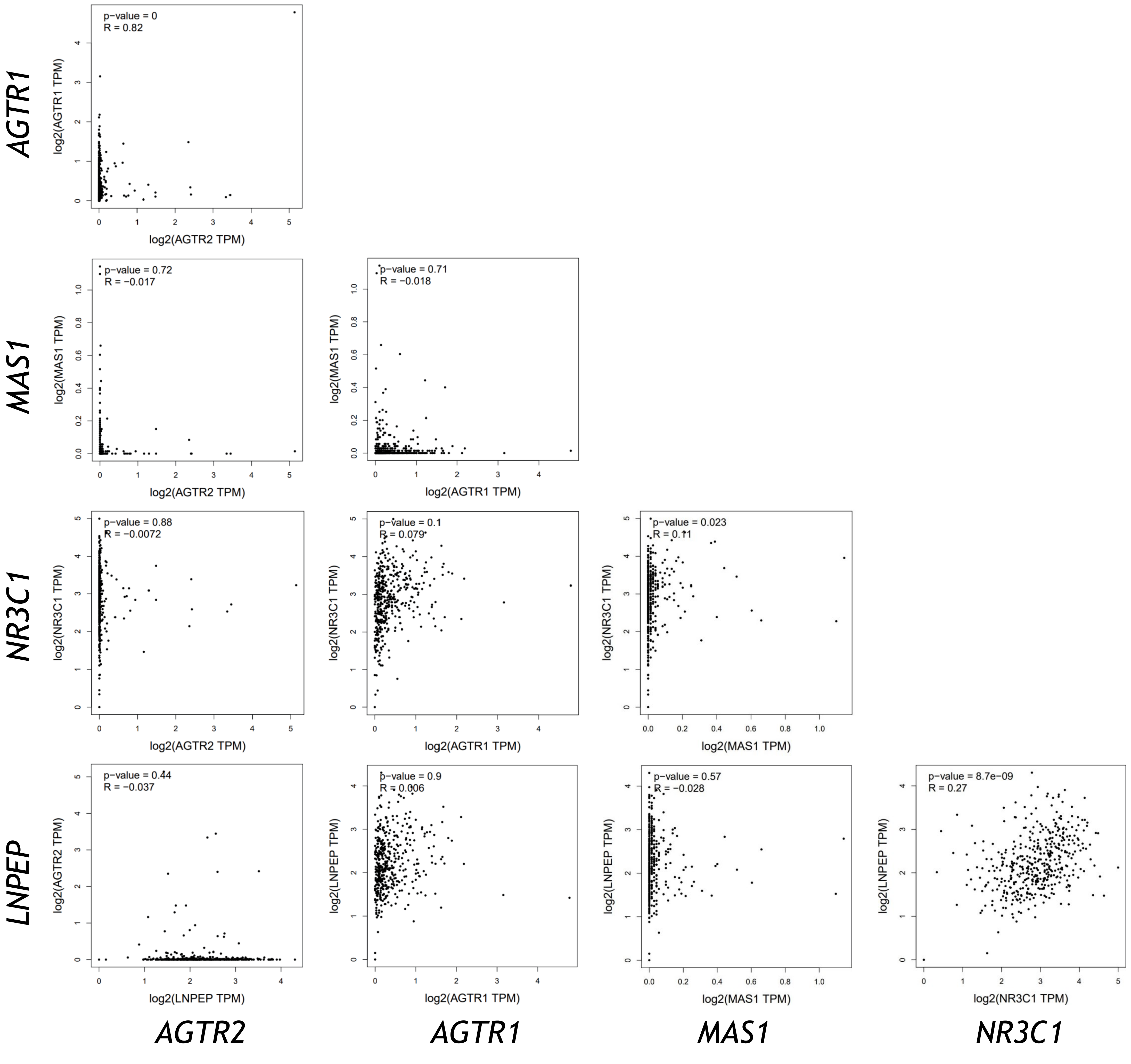

Supplementary Figure S6. Correlation analysis of gene expression in ovarian cancer. Scatter plots illustrating pairwise correlations between the indicated genes in ovarian cancer samples from the TCGA-OV cohort. Analyses were performed using the GEPIA platform, based on data from The Cancer Genome Atlas. Pearson correlation coefficients (R) and corresponding p-values are shown on each plot. Non-log-transformed expression values were used for statistical calculations, while log-scale transformation was applied for data visualization.
